# PEDOT:PSS for Printed Flexible Sensors: Linking Film Morphology to Transduction, Stability and Calibration

**DOI:** 10.3390/polym18212775

**Published:** 2026-11-06

**Authors:** Fatih Gül, Erkan Caner Ozkat, Karim El Haloui

**Affiliations:** 1 Department of Electrical and Electronics Engineering, Faculty of Engineering and Architecture, Recep Tayyip Erdogan University, 53100 Rize, Turkey; fatih.gul@erdogan.edu.tr; 2 Artificial Intelligence and Internet of Things Research Laboratory, Faculty of Engineering and Architecture, Recep Tayyip Erdogan University, 53100 Rize, Turkey; erkancaner.ozkat@erdogan.edu.tr; 3 Department of Mechanical Engineering, Faculty of Engineering and Architecture, Recep Tayyip Erdogan University, 53100 Rize, Turkey; 4 International Digital Laboratory, Warwick Manufacturing Group, University of Warwick, Coventry CV4 7AL, UK

**Keywords:** PEDOT:PSS, secondary doping, organic electrochemical transistors, sensor drift, limit of detection

## Abstract

Poly(3,4-ethylenedioxythiophene):poly(styrene sulfonate) (PEDOT:PSS) is the most widely used conducting polymer in printed and flexible sensors. It is water-processable, can be made compliant, and conducts holes and ions. Most of these properties, and its drift, respond to one variable, the grain morphology of the film, around which this review is organised. The review first describes PEDOT-rich grains in a PSS matrix and the resulting mixed ionic–electronic transport. It then compares secondary doping, post-treatments, ionic additives, and crosslinkers by their effects on conductivity, stretchability, and aqueous stability. Printing routes, inks, application-specific device stacks, and transduction modes follow. Next, failure mechanisms are mapped onto the sensor classes they dominate. Reversible water uptake, ageing, electrochemical de-doping, cracking, delamination, and loss of skin contact produce different drift signatures and need different corrections. Analytical performance is compared across material classes. As a bare chemiresistor, the polymer works in the parts-per-million decade. As the channel of an electrochemical transistor with a functionalised gate, it reaches nanomolar detection limits. Detection limit, linear range, and interference at a stated humidity are rarely reported together. This review documents that gap. Finally, it describes the calibration steps that recover a measurand from a drifting signal and proposes a minimum reporting set.

## 1. Introduction

Printed and flexible electronics has found its clearest application in sensing. A sensor must be in contact with what it measures, and many targets, such as skin, textiles, and packaging, are soft, curved, wet, or moving. On such targets, conformability and low cost per unit area decide whether a measurement is possible [1,2,3]. Conducting polymers meet these requirements because one solution-processed film can act as electrode, transducer, and mechanical layer [4,5,6].

Among conducting polymers, poly(3,4-ethylenedioxythiophene):poly(styrene sulfonate) (PEDOT:PSS) has become the default choice for printed devices [7,8,9]. It is supplied as a stable aqueous dispersion, it is air-stable in the doped state, and its thin films are transparent [10,11]. Moreover, it conducts ions as well as holes, so it can transduce ionic and biological signals directly [12,13]. Polyaniline (PANI) and polypyrrole remain useful for specific chemistries, but their processability and stability are more limited [14,15].

The properties that make PEDOT:PSS useful respond largely to one structural variable. A PEDOT:PSS film consists of PEDOT-rich grains in a PSS-rich matrix, and the size, shape, and connectivity of these grains set its behaviour [16,17,18]. The same morphology sets conductivity, ductility, water uptake, and ionic access. Processing therefore changes these properties together. For instance, an additive chosen to raise conductivity also changes humidity sensitivity and long-term drift [19,20]. However, device studies usually report the first effect and seldom the others.

Several reviews already cover PEDOT:PSS, and this review is designed to complement them rather than to repeat them. Table 1 compares the scope of ten representative reviews with the scope of this one. The Processing column includes conductivity control, and the Mechanics column includes stretchability. The Printing column includes ink formulation, the Stability column includes ageing and drift, and the Calibration column includes drift compensation and unit-to-unit transfer. Three observations follow from that comparison.

First, the existing literature is organised by application. Conductivity enhancement [21,22], stretchability [8,9], bioelectronic interfaces [23,24], energy conversion [11], and thermoelectrics [25] each have an authoritative treatment. Hence, a reader who seeks the highest reported conductivity, or the most stretchable formulation, is well served.

**Table 1:** Scope of representative PEDOT:PSS reviews and of this review.

Review	Primary Axis	Processing	Mechanics	Bioelectronics	Printing	Stability	Calibration
Groenendaal et al. 2000 [7]	PEDOT chemistry and derivatives	✔	–	(✔)	–	–	–
Ouyang 2013 [22]	Secondary doping	✔	–	–	–	–	–
Shi et al. 2015 [21]	Conductivity enhancement	✔	(✔)	–	–	(✔)	–
Sun et al. 2015 [11]	Energy conversion and storage	✔	–	–	(✔)	(✔)	–
Wen and Xu 2017 [10]	Water-processable formulations	✔	(✔)	–	(✔)	–	–
Kayser and Lipomi 2019 [8]	Stretchable conductors	✔	✔	(✔)	(✔)	–	–
Fan et al. 2019 [9]	Flexible and stretchable devices	✔	✔	(✔)	(✔)	(✔)	–
Donahue et al. 2020 [23]	Bioelectronic interfaces	✔	(✔)	✔	–	(✔)	–
Panigrahy and Kandasubramanian 2020 [25]	Thermoelectrics	✔	–	–	(✔)	(✔)	–
Seiti et al. 2023 [24]	Bioelectronic applications	✔	✔	✔	(✔)	(✔)	–
This review	Morphology as the shared variable, followed to a calibrated measurement	✔	✔	✔	✔	✔	✔

A tick (✔) marks a topic that the review treats as a main axis. A tick in parentheses, (✔), marks a topic that the review touches on without treating it in depth. A dash marks a topic outside the scope that the review states for itself.

Second, none of the ten reviews treats long-term stability and drift, or the calibration layer that converts a drifting signal into a measurement, as a main axis. Six of the ten touch on stability, and none treats calibration. A third topic, reporting practice, is treated by none of the listed reviews and therefore has no column. These are not peripheral topics. Drift, rather than sensitivity, often limits the useful life of a chemical sensor [26]. Moreover, a figure of merit quoted without the humidity at which it was measured cannot be compared with any other.

Third, and the reason for organising this review around morphology, the application-centred reviews necessarily repeat the same material science in different contexts. Grain morphology sets conductivity, ionic access, mechanics, and water uptake simultaneously. Consequently, a treatment chosen in a stretchability review and one chosen in a bioelectronics review move the same variable with opposite priorities. Organising the literature around that single variable makes the trade-offs visible as trade-offs rather than as independent results. Furthermore, this organisation is what makes the stability, calibration, and reporting sections of this review possible.

Organising a review around one variable carries a risk that should be stated at the outset. Morphology is used here as the variable to which the largest number of sensor properties respond, not as their sole cause. The oxidation level of the PEDOT phase, the PEDOT-to-PSS ratio, and the accessibility of the film to ions act in parallel with morphology. The resistance of the printed contacts and the architecture of the device do so as well, and in electrochemical devices these two can dominate. Where a link between a morphological variable and a sensor metric has been established by measuring both on the same films, this review says so. Where the link is a proposed explanation that is consistent with the data but not demonstrated by it, the review says that instead. Table 2 carries that distinction explicitly.

This review follows the chain from film morphology to a calibrated measurement for a single polymer, and two boundaries define its scope. First, the review covers PEDOT:PSS and blends in which PEDOT:PSS is the active conductor. Other conducting polymers and filler composites appear only as reference points. Second, the review concentrates on printed or solution-processed films on flexible substrates.

The remainder of this review is structured as follows. Section 2 describes the structure of PEDOT:PSS films and their mixed ionic–electronic transport. Section 3 compares the treatments that control morphology. Section 4 reviews printing routes, inks, and device stacks. Section 5 compares the transduction modes and their analytical performance. Section 6 treats humidity, ageing, fatigue, and washing as sources of drift and maps them onto the sensor classes they dominate. Section 7 describes the calibration steps that recover a measurand from a drifting signal. Section 8 proposes a minimum reporting set. Section 9 then examines these findings together as three recurring trade-offs. Section 10 states open problems and the limitations of this review. Finally, Section 11 summarises the main findings.

## 2. Structure and Charge Transport in PEDOT:PSS

This section describes the material in five parts. The first part covers grain morphology and electronic transport, the second ionic transport and water uptake, and the third mechanical behaviour. The fourth part maps each sensor type onto the morphological variable that governs its figure of merit. The fifth part separates the features specific to PEDOT:PSS from those shared with other conductors made of connected domains.

**Figure 1:**
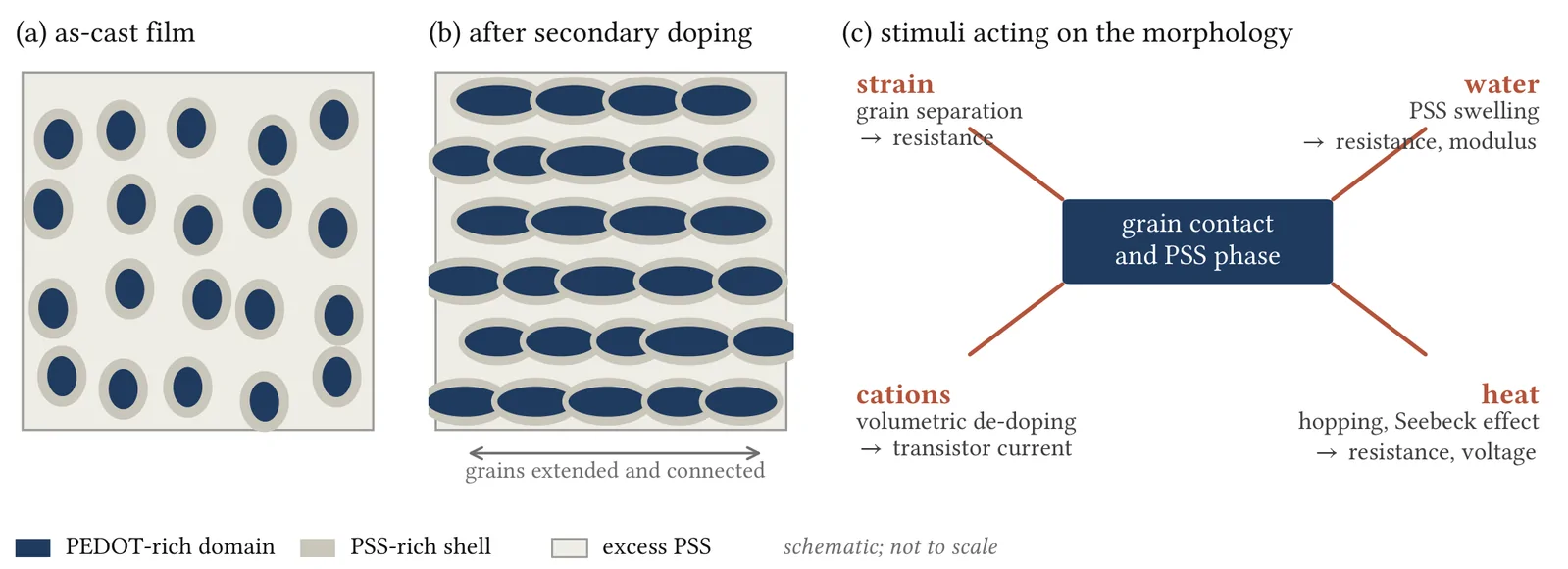
Simplified conceptual model of PEDOT:PSS film morphology and the stimuli acting on it.

Figure 1 summarises the morphology and the stimuli that act on it. The panels are schematic rather than representations of measured microstructure. Grain size, shape, shell thickness, and packing are drawn for clarity and are not to scale, and a real film is disordered in three dimensions. In this model, PEDOT-rich grains are embedded in a PSS-rich matrix, and the PSS shells between grains dominate the film resistance. Water, solvents, ions, heat, and strain all change the same structure. Hence, these stimuli cannot be treated as independent variables. Table 2 states which sensor metric each change governs.

### 2.1. Grain Morphology and Electronic Transport

PEDOT is a polythiophene that is insoluble in its doped state. Polymerisation in the presence of PSS yields a water-dispersible complex, in which PSS acts as both counter-ion and dispersant [7,11]. Commercial grades differ in their PEDOT-to-PSS ratio, and conductivity falls as the PSS fraction rises [27]. Stöcker et al. [27] combined impedance spectroscopy with thermoelectric measurements on one series of formulations spanning ratios from 1:1 to 1:30. They found the carrier density to be independent of the PSS content. Furthermore, they described the conductivity as percolation between conducting PEDOT:PSS complexes embedded in a matrix of excess PSS. The conductivities of the complexes and of the matrix were about 2.3 S cm^−1^ and $10^{-3}$ S cm^−1^, respectively. However, this finding is quoted here for the system in which it was obtained and should not be read as a general rule. It describes a dilution series at a fixed oxidation level, whereas deliberate oxidation or reduction of the PEDOT phase does change the carrier density. This change is the basis of thermoelectric operation (Section 5).

In a cast film, PEDOT-rich grains are surrounded by shells of insulating PSS [16,17]. Charge moves easily within a grain and with difficulty across the shells between grains. The shells therefore dominate the film resistance, and as-cast films typically conduct about 1 S cm^−1^ or less [21,28]. Spin-cast films are also anisotropic, because the grains flatten into domains parallel to the substrate [17].

Treatments that thin the shells, or that order PEDOT into crystalline domains, raise conductivity by orders of magnitude [18,29]. Spectroscopic studies attribute this rise to morphology and doping level rather than to a change in the polymer itself [16,30].

### 2.2. Mixed Ionic–Electronic Conduction and Water Uptake

PSS is a hygroscopic polyelectrolyte, so a PEDOT:PSS film absorbs water from the air [31,32]. The two carriers, holes and cations, follow separate routes. Holes move along the connected PEDOT-rich domains, which form a percolating electronic network whose bottleneck is the PSS shell between grains. In contrast, cations move through the hydrated, swollen PSS-rich regions, which form a separate ionic network whose bottleneck is the degree of hydration. The two networks interpenetrate but remain physically distinct phases. The term mixed conductor describes the composite rather than a single phase in which both carriers are mobile in the same way [12,13]. However, the coupling between the two networks is electrochemical and occurs at their boundary. A cation that enters the hydrated phase compensates a sulfonate site, and the hole that the site previously stabilised is removed from the adjacent PEDOT chain. Therefore, ions can enter the bulk of the film and change the doping level of PEDOT throughout its volume rather than only at a surface [12,13]. The resulting volumetric capacitance is much larger than the double-layer capacitance of a planar metal electrode [33,34]. The structure that favours ionic transport differs from the one that favours electronic transport. Processing therefore trades one against the other [12].

For sensing, this coupling has two consequences. First, it is the operating principle of organic electrochemical transistors (OECTs) and of low-impedance skin electrodes (Section 5). Second, it is the reason why humidity changes the resistance of every PEDOT:PSS device, including devices intended to measure strain or temperature (Section 6).

### 2.3. Mechanical Behaviour

The mechanical properties of PEDOT:PSS also depend on water and processing. Pristine films are stiff and brittle, with moduli of the order of gigapascals [35], and they remain stretchable only up to about 10% strain [8]. Absorbed water softens the film [35]. Annealing removes residual water, and the loss of this water changes the PSS phase and the grain contacts [36].

Plasticising additives such as ionic liquids, surfactants, and polyols raise the strain at which the film cracks [37,38,39]. Moreover, supramolecular network designs now combine high conductivity with high crack-onset strain under physiological conditions [40].

### 2.4. From Morphology to Sensor Metrics

The statement that morphology sets several properties at once is useful only if it can be read in the opposite direction. In that direction, the question is which morphological variable decides the figure of merit of a given sensor type. Table 2 states that mapping, which is the organising claim of the rest of this review. The Basis column states how the link is supported. The entry Measured means that the cited work determined the morphological variable and the metric on the same films. The entry Inferred means that the link is a proposed explanation consistent with the data but not demonstrated by a joint measurement. The final column names the penalty that comes with optimising the metric. The symbol $\mu C^{*}$ denotes the product of hole mobility and volumetric capacitance.

Three points in the table merit attention, because they mark where the mapping is not symmetric.

First, a piezoresistive sensor depends on the weakest part of the structure. Its gauge factor arises from the inter-grain contacts that treatments are designed to improve, so the processing that maximises conductivity also flattens the strain response. Highly treated films make poor strain sensors and good interconnects, which is the same fact seen from two directions.

Second, an OECT depends on the coupling between the two transport networks. Its transconductance scales with the product of hole mobility and volumetric capacitance [34], that is, with the product of an electronic and an ionic property. Treatments that order PEDOT raise the first and can lower the second by closing the hydrated PSS pathways. Hence, the optimum is interior rather than at either extreme.

Third, a chemiresistor depends on accessibility. Its response requires analyte molecules to reach PEDOT sites and change the doping level. Consequently, the same open, hydrated, PSS-rich structure that depresses conductivity raises the chemical response. This structure also makes the device sensitive to water (Section 6.1).

Overall, the grain morphology of PEDOT:PSS sets its electronic conductivity, its ionic access, and its mechanical response at once. Table 2 states which of these properties a given sensor type depends on.

**Table 2:** Morphological variables of PEDOT:PSS films and the sensor metrics they govern.

Sensor Type	Governing Morphological Variable	Metric It Sets	Basis	Penalty of Optimising It
Piezoresistive strain and pressure	Inter-grain contact density and crack network	Gauge factor ↓ with PSS removal and grain connection	Inferred	Treatments that raise conductivity flatten the strain response; cracking raises sensitivity and destroys linearity
Humidity	Volume and accessibility of the hydrated PSS phase	Response magnitude ↑ with PSS fraction	Inferred	The same structure makes every other mode humidity sensitive
Temperature	Activation energy of inter-grain hopping	Temperature coefficient ↓ with crystalline order	Inferred	Needs a humidity barrier, which slows the thermal response
Chemiresistive gas	Open porosity and analyte access to PEDOT sites	Response ↑ with accessible, hydrated structure	Inferred	Low intrinsic selectivity and strong humidity cross-sensitivity
Electrochemical transistor	Coupling of hole mobility and volumetric capacitance	Transconductance ↑ with $\mu C^{*}$ [34]	Measured [20,34]	Swelling and delamination; crosslinking stabilises but raises impedance [41]
Electrophysiological electrode	Volumetric capacitance at the hydrated interface	Impedance ↓ with ionic access [33]	Measured [42]	Drying ends the low-impedance state; ionic gels trade shelf life for it [43]
Thermoelectric	Oxidation level of the PEDOT phase	Seebeck ↓, conductivity ↑ with doping [44,45]	Inferred	The power factor has an interior optimum; de-doping drifts in air

An upward arrow (↑) means that the metric rises as the quantity named after “with” increases. A downward arrow (↓) means that it falls.

### 2.5. What Is Specific to PEDOT:PSS and What Is Not

Sensor response governed by microstructure is not peculiar to PEDOT:PSS. Separating the general mechanism from the specific one keeps the organising claim of this review within its evidence.

The general part is percolation and access. In any conductor made of connected domains, the response to a mechanical or chemical stimulus is dominated by the weakest junctions along the conducting path. In addition, the internal area that a stimulus can reach decides how much of the material participates. Laser-induced graphene (LIG) is the clearest contemporary demonstration. As reviewed by Movaghgharnezhad and Kang [46], a single laser step converts a carbon-rich polymer surface into a three-dimensional porous carbon. The laser parameters tune pore structure, sheet resistance, and specific surface area, which then set the performance of the electrochemical and physical sensors built from it. Printed carbon nanotube (CNT) films and their composites with PEDOT:PSS [47] are further sensors made of connected domains. The same holds for graphene–PEDOT:PSS films [48] and for enzymatic electrodes written into polyimide [49,50].

Three features are specific to PEDOT:PSS. First, its microstructure is not an inert scaffold. It is defined by a soluble, hygroscopic second component that stays in the film. Hence, the variable that controls conduction is the same variable that controls water uptake. By contrast, the pore structure of a laser-written carbon does not change with relative humidity (RH) in the way a PSS phase does. Second, the PSS phase conducts ions. The film is therefore a mixed conductor rather than a porous electronic conductor, and its capacitance is volumetric rather than interfacial. Third, the microstructure remains reconfigurable after deposition, by a solvent, an acid, or an anneal (Section 3). A laser-written structure, however, is fixed when it is written.

The practical consequence appears at bioelectronic interfaces (Section 5.5). There, porous carbon and PEDOT:PSS answer to different figures of merit. Carbon answers through electrochemically accessible area and charge injection, whereas PEDOT:PSS answers through a capacitance distributed throughout the volume of the film. Section 3 examines how processing controls that volume.

In summary, this section has traced the structure of PEDOT:PSS from its grains to its sensor metrics. Holes and cations move through separate networks that couple electrochemically at their boundary. Water and processing alter this structure, and with it the electronic, ionic, and mechanical properties of the film. Each sensor type depends on a particular morphological variable, and optimising its metric carries a penalty. Percolation and access are shared with other conductors made of connected domains. However, the hygroscopic, ion-conducting PSS phase and the reconfigurable microstructure are specific to PEDOT:PSS.

## 3. Morphology Control by Secondary Doping and Post-Treatment

Processing changes the morphology of PEDOT:PSS more than any other variable. This section reviews the morphology-control treatments in six parts. The first four subsections compare four groups of treatments: polar solvents and polyols, acid and alcohol post-treatments, ionic additives and surfactants, and crosslinkers and hydrogels. The fifth subsection states the trade-off that these groups share, and Table 3 summarises the comparison. The sixth subsection shows that conductivity is not a device metric, using studies that measured material and device properties on the same films.

### 3.1. Polar Solvents and Polyols

The most widely used treatment adds a high-boiling polar solvent to the dispersion. Dimethyl sulfoxide (DMSO) and ethylene glycol are the standard choices [21,22]. These solvents screen the Coulombic interaction between PEDOT and PSS, allow the PEDOT chains to extend, and separate part of the excess PSS. Solvents can also be applied as a post-treatment. For instance, Kim et al. [28] obtained 1418 S cm^−1^ in electrode films in this way. Moreover, solvent addition changes the work function and surface composition of the film [51].

Polyols such as sorbitol act in a similar way and also plasticise the film [52]. Nardes et al. [19] showed that sorbitol treatment alters the environmental stability and work function of the film as well as its conductivity. Similarly, Okuzaki et al. [53] treated wet-spun fibres with ethylene glycol and showed that the same chemistry applies to one-dimensional structures.

### 3.2. Acid and Alcohol Post-Treatments

Post-treatment of a cast film can remove more PSS than an additive can. For instance, treatment with sulfuric acid produced films with conductivities above 3000 S cm^−1^ [54]. Kim et al. [29] formed ordered PEDOT nanofibrils with still higher conductivity through solution-processed crystallisation. Crystalline PEDOT domains, which also form under ethylene glycol treatment, explain part of the gain [18,29]. Notably, Alemu et al. [55] reached conductivities of the order of $10^{3}$ S cm^−1^ without acid, using a simple methanol treatment.

These treatments act on the finished film. However, strong acids attack many flexible substrates and printed electrodes. Acid routes therefore suit stand-alone electrodes better than integrated sensors with several printed layers.

### 3.3. Ionic Additives and Surfactants

Stretchable sensors require a conductivity that survives strain. Ionic additives can act as secondary dopants and plasticisers at the same time. For instance, Wang et al. [38] used ionic additives to obtain films that remained highly conductive beyond 100% tensile strain. Kee et al. [56] showed that ionic liquids also control the molecular ordering of PEDOT in aqueous formulations. Similarly, Teo et al. [39] raised the stretchability of transparent PEDOT:PSS electrodes with ionic liquids.

In addition, Vosgueritchian et al. [37] showed that a fluorosurfactant lowered the sheet resistance by 35% and allowed PEDOT:PSS to wet hydrophobic poly(dimethylsiloxane) (PDMS). Lipomi et al. [57] demonstrated that the surface treatment of the PDMS and a fluorosurfactant additive together determine how the film resistance responds to stretching. Intrinsically stretchable PEDOT films and hybrids with silver nanowires extend the working range further [58,59].

### 3.4. Crosslinkers and Hydrogels

Uncrosslinked PEDOT:PSS films swell and can delaminate in water. The silane crosslinker 3-glycidoxypropyltrimethoxysilane (GOPS) is the usual remedy in electrochemical and biological devices [41]. ElMahmoudy et al. [41] showed that GOPS improves aqueous stability and mechanical strength but raises the electrochemical impedance. Hence, its optimum content depends on which property is targeted. Moreover, crystallinity and composition govern the long-term stability of transistor channels in water [20].

Pure PEDOT:PSS hydrogels represent the opposite extreme. Lu et al. [60] obtained hydrogels with tissue-like softness from interconnected nanofibril networks formed with DMSO and controlled dry annealing. Such hydrogels maximise ionic access, which is useful at tissue interfaces.

### 3.5. The Conductivity–Stability Trade-Off

Conductivities in Table 3 are the values stated by the cited work for each treatment, not best-in-class figures. The last column states the main message of the table. The treatment that maximises conductivity is not the treatment that a given sensor needs.

Table 3 shows a pattern common to all four groups. Treatments that raise conductivity do so by removing or rearranging PSS. However, the remaining PSS still absorbs water, and plasticised films swell more readily. High conductivity therefore does not imply a stable baseline.

For an interconnect, conductivity is the target. For a sensor, however, the baseline must also remain stable against humidity, temperature, and time. These requirements favour different treatments. Therefore, the treatment should be selected for the measurand rather than for the highest reported conductivity.

**Figure 2:**
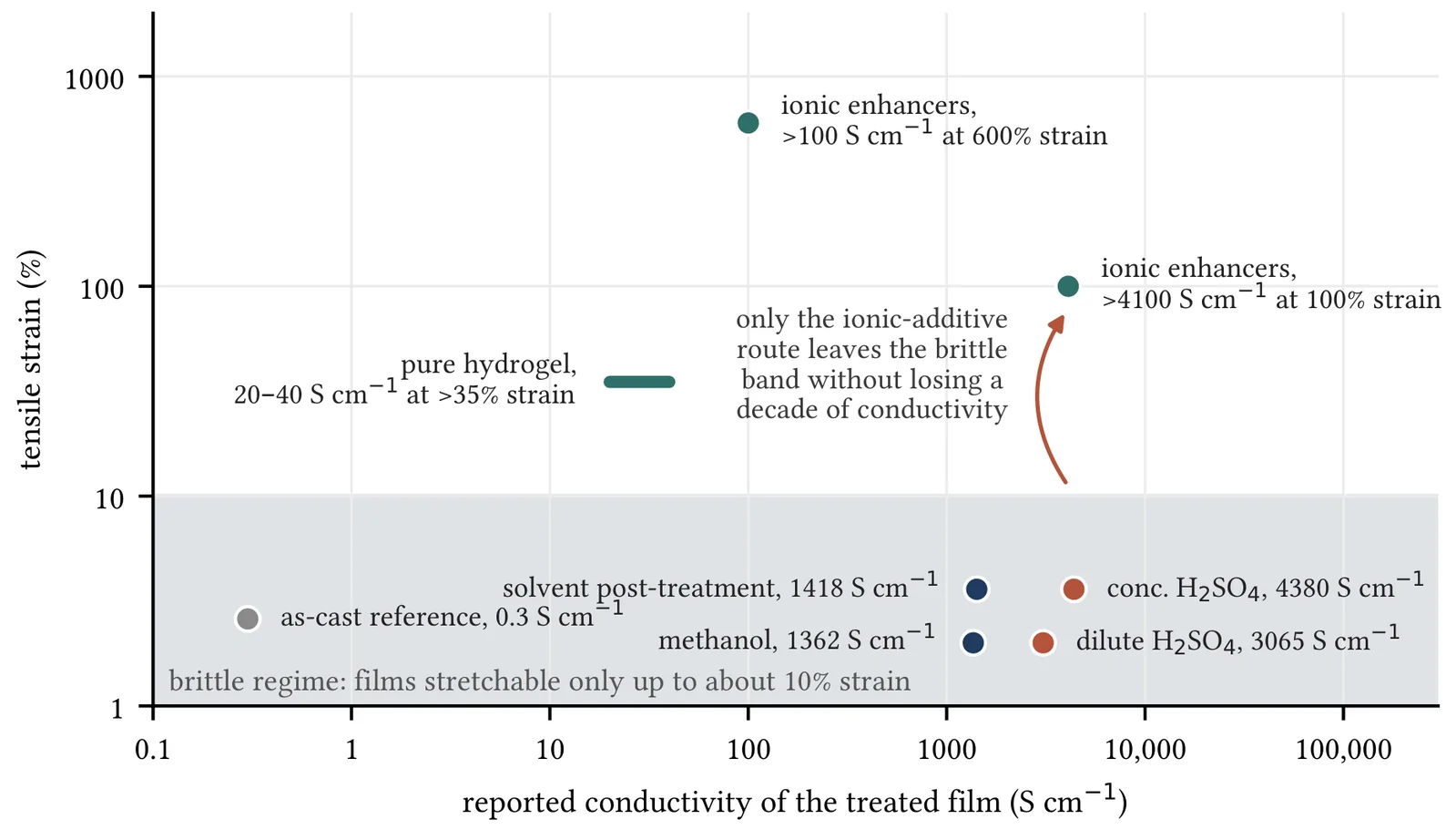
Tensile strain against reported conductivity of treated PEDOT:PSS films.

Three cautions apply to any comparison of this kind, and they apply to Table 3 and Figure 2 as much as to the literature they summarise. First, reported conductivities come from different commercial grades, which differ in PEDOT-to-PSS ratio before any treatment is applied [27]. Second, they come from films of different thickness deposited by different routes. A four-point measurement on a spun film of some tens of nanometres differs from one on a screen-printed layer an order of magnitude thicker. Third, they are measured in laboratory air whose RH is usually not stated. For a hygroscopic film, that humidity changes the number itself (Section 6.1). Therefore, the table is a map of what each route does to the film rather than a ranking. Accordingly, the arithmetic difference between two entries carries less information than the direction in which each route moves the film.

Figure 2 places each treatment by its reported conductivity (horizontal axis) and by tensile strain (vertical axis). Each point is a value stated by the cited work for that treatment, not a best-in-class figure. The points cover solvent post-treatment [28], methanol [55], dilute and concentrated sulfuric acid [29,54], ionic enhancers [38], and pure hydrogels [60]. An as-cast reference of 0.3 S cm^−1^, the starting value in two of these works, is added for comparison [54,55]. The shaded band collects the treatments that leave a brittle film. Points in this band were reported on films that remain stretchable only up to about 10% strain [8]. The strain coordinate of those points is therefore the crack-onset regime rather than a measured value at that conductivity. Outside the band, the strain coordinate is the value stated by the source.

**Table 3:** Morphology-control treatments for PEDOT:PSS films.

Treatment and Agent	Reported Conductivity	Mechanical and Aqueous Behaviour	Suited to/Unsuited to
Polar solvent additive or post-treatment: DMSO, ethylene glycol [22,28]	1418 S cm^−1^ after solvent post-treatment [28]	Film remains brittle unless a plasticiser is added	Suited: printed interconnects, electrodes. Unsuited: strain sensing, where it flattens the response
Polyol additive: sorbitol [19,52]	Conductivity gain with plasticisation; value not stated for a common reference film	Greater ductility; altered environmental stability [19]	Suited: flexible electrodes needing some compliance. Caution: environmental stability changes with the additive
Acid post-treatment: dilute H_2_SO_4_ [54]; concentrated H_2_SO_4_ [29]	Dilute: 0.3 → 3065 S cm^−1^, 39 Ω sq^−1^ at 80% transmittance [54]. Concentrated: 4380 S cm^−1^, 46 Ω sq^−1^ at 90% [29]	Crystalline nanofibrils; aggressive to substrates and to printed layers	Suited: stand-alone transparent electrodes. Unsuited: multilayer printed sensors, most plastic substrates
Alcohol post-treatment: methanol [55]	0.3 → 1362 S cm^−1^ [55]	Mild conditions; PSS removed, morphology changed	Suited: finished films on plastic where acid cannot be used
Ionic additive: ionic liquids and ionic enhancers [38,39,56]	>3100 S cm^−1^ unstrained; >4100 at 100% strain; 3600 after 1000 cycles to 100%; >100 at 600%, fracture at 800% [38]	Conductive far beyond the crack-onset strain of pristine films	Suited: stretchable interconnects and skin-mounted devices. Caution: mobile ions can migrate and drift
Surfactant: fluorosurfactant [37]	Sheet resistance lowered by 35%; 46 Ω sq^−1^ at 82% transmittance [37]	Enables wetting of hydrophobic PDMS	Suited: printing directly on elastomers; multilayer stacks
Crosslinker: GOPS [41]	Conductivity decreases with crosslinker content	Aqueous stability and mechanical strength up; electrochemical impedance up [41]	Suited: OECTs and devices operated in water. Unsuited: low-impedance electrodes, where low impedance is the figure of merit
Hydrogel formation: DMSO plus dry annealing and rehydration [60]	∼20 S cm^−1^ in buffered saline and ∼40 S cm^−1^ in water [60]	>35% strain, Young’s modulus ∼2 MPa [60]	Suited: tissue interfaces needing softness and ionic access. Unsuited: dry-film sensors, where swelling moves the baseline
Thermal annealing: temperature and time [36,51]	Sets the starting point for every route above	Water removal; altered grain contact [36]	Caution: the baseline depends on thermal history, so the anneal must be reported with the formulation

The solvent, alcohol, and acid routes all sit in the shaded band. The ionic-additive route is the only one that leaves the band without losing a decade of conductivity. In contrast, the hydrogel route trades two decades of conductivity for softness and ionic access. A designer who reads the figure horizontally is choosing an interconnect, whereas one who reads it vertically is choosing a sensor.

### 3.6. Conductivity Is Not a Device Metric

Beyond the three cautions stated above, the quantity the field optimises is not the quantity a device delivers. Four statements make this concrete. Table 4 collects the studies that support them. The study behind each of its first five rows measured a material property and a device property on the same films. The last row is a counter-example. Entries are the values stated by the cited work, and a dash means that the quantity was not reported there. The table is therefore a selection on that criterion, not a survey.

First, a higher electronic conductivity does not imply a lower electrochemical impedance. For instance, ElMahmoudy et al. [41] showed that GOPS crosslinking raises the electrochemical impedance while the film remains highly conducting. The crosslinker also improves aqueous stability and mechanical integrity. Their transistors containing 1 wt% GOPS remained steady over three weeks. Conduction through the PEDOT network and ion exchange across the film–electrolyte boundary are different transport problems, and a single additive can move them independently.

Second, a higher conductivity does not imply a better transistor. Rivnay et al. [42] showed that ion uptake into PEDOT:PSS gives a purely volumetric capacitance of 39 F cm^−3^. Hence, transconductance can be raised by thickening the channel at unchanged conductivity. Crystalline order is another route to a higher transconductance. Kim et al. [20] reported a transconductance of about 20 mS and a volumetric capacitance of 113 F cm^−3^. The figure of merit $\mu C^{*}$ reached about 490 F cm^−1^ V^−1^ s^−1^. The films showed marginal swelling after more than 20 days of water immersion and more than 2000 switching cycles.

Third, a higher conductivity does not imply a mechanically usable film. Lipomi et al. [57] compared PEDOT:PSS films cast on PDMS after two surface treatments. On oxygen-plasma-treated PDMS, the resistivity rose by four orders of magnitude at only 10% strain. On PDMS treated with ultraviolet (UV) light and ozone, with 1% fluorosurfactant added to the dispersion, the resistance only doubled at 50% strain. The governing variables there are the surface treatment and the wetting additive, not the conductivity of the film.

Fourth, a higher conductivity does not imply less drift. Every route to high conductivity discussed above removes or rearranges the water-absorbing PSS phase but leaves part of it in the film. Notably, the one entry in Table 4 that reports a baseline stable across a humidity range obtains it from a passivation bilayer rather than from the conductor. Tachibana et al. [61] reported a sensitivity of −0.77% °C^−1^ between 25 and 50 °C, stable from 30% to 80% RH.

The last row of Table 4 is the counter-example that shows the size of the gap. Wang et al. [38] characterised conductivity over the widest strain range in that table. They reported it at 0%, 100%, and 600% strain. They also reported it after 1000 cycles and up to fracture at 800%. However, this characterisation describes an interconnect. No sensing metric accompanies it, and none follows from it.

In summary, secondary doping, post-treatment, ionic additives, and crosslinkers all act on the same PSS phase. Each choice moves conductivity, stretchability, and aqueous stability together, and conductivity alone predicts neither of the other two. Section 4 considers the further constraints that printing adds.

## 4. Printing PEDOT:PSS

Printing turns a formulation into a device and adds its own constraints. This section treats the process in five parts: ink formulation, printing routes, fibre and three-dimensional formats, the device stack, and print-to-print variation.

### 4.1. Ink Formulation

Each printing route accepts a narrow window of viscosity and surface tension. Inkjet printing requires a viscosity of a few to a few tens of millipascal seconds. In contrast, screen printing requires shear-thinning pastes that are several orders of magnitude more viscous [62,63]. Moreover, the jetting waveform sets droplet size and the window for stable droplet ejection [64]. The rheology of PEDOT:PSS inks also depends on concentration. One low-viscosity inkjet ink was reported to behave as a Newtonian liquid [64], whereas concentrated inks for direct writing are shear thinning [65]. When a shear-thinning ink is delivered by a pulsed actuator, its flow is a pulsating non-Newtonian flow. Pulsating laminar flow of power-law fluids has been solved analytically for plane and concentric annular ducts [66,67]. In the plane duct, the friction factor depends on the power-law index as well as on the amplitude and frequency of the pulsation [66]. The pressure needed to deliver such an ink by pulsed actuation may therefore differ from the steady-flow value predicted with the same viscosity law.

Formulation additives also serve two purposes at once. Solvents and surfactants adjust printability, and the same compounds act as secondary dopants (Section 3). Consequently, a change made for printability also changes the electrical and humidity response of the printed film. Fluorosurfactants are a clear case, since they improve wetting and also lower sheet resistance [37].

### 4.2. Printing Routes

Inkjet printing is the most common digital route for PEDOT:PSS. It has produced transport layers for organic solar cells, temperature-sensor arrays, and tattoo electrodes for electrophysiology [68,69,70]. Seekaew et al. [48] reported inkjet-printed graphene–PEDOT:PSS ammonia sensors on flexible film. Similarly, Kuzubasoglu et al. [47] demonstrated inkjet-printed CNT/PEDOT:PSS films on textiles as temperature sensors.

Aerosol jet printing decouples ink viscosity from nozzle size and permits printing on non-planar substrates [71]. Tarabella et al. [72] printed PEDOT:PSS lines on flexible Kapton and related their width and thickness to the ratio of sheath to carrier gas flow. Screen printing and roll-to-roll gravure are the routes to large area and high volume [73,74]. However, they require thicker formulations, so the ink must often be reformulated between prototyping and volume production. Table 5 summarises the routes. Its feature sizes and formulation windows are indicative ranges from the cited reports, not specifications.

### 4.3. Fibres, Hydrogels, and Three-Dimensional Structures

PEDOT:PSS is not limited to thin films. Wet spinning produces conductive fibres, and post-treatment in ethylene glycol raises their conductivity [53]. Moreover, polyurethane/PEDOT:PSS composite fibres combine elastomeric stretch with conductivity and respond to strain. Their electromechanical behaviour under tensile and cyclic stretching has been modelled [75]. Beyond fibres, Yuk et al. [65] formulated a PEDOT:PSS ink for direct three-dimensional printing at high resolution. This route links printing to the pure PEDOT:PSS hydrogels of Section 3 [60]. In addition, water-enabled healing of PEDOT:PSS films offers a route to repair mechanical damage [76].

### 4.4. Substrates, Encapsulation, and the Device Stack

The substrate sets the strain that the film experiences and the temperature at which it can be annealed. Polyimide tolerates high annealing temperatures, whereas polyethylene terephthalate is less expensive. PDMS and polyurethane provide skin conformality [77,78], and paper and nanocellulose offer disposable substrates [79]. However, bending strains every layer of the stack. In flexible oxide transistors, the channel cracks above a critical strain, and a thinner substrate reduces the critical bending radius [80]. In flexible organic solar cells, weak adhesion between the hole-transport and active layers limits bending durability [81].

**Figure 3:**
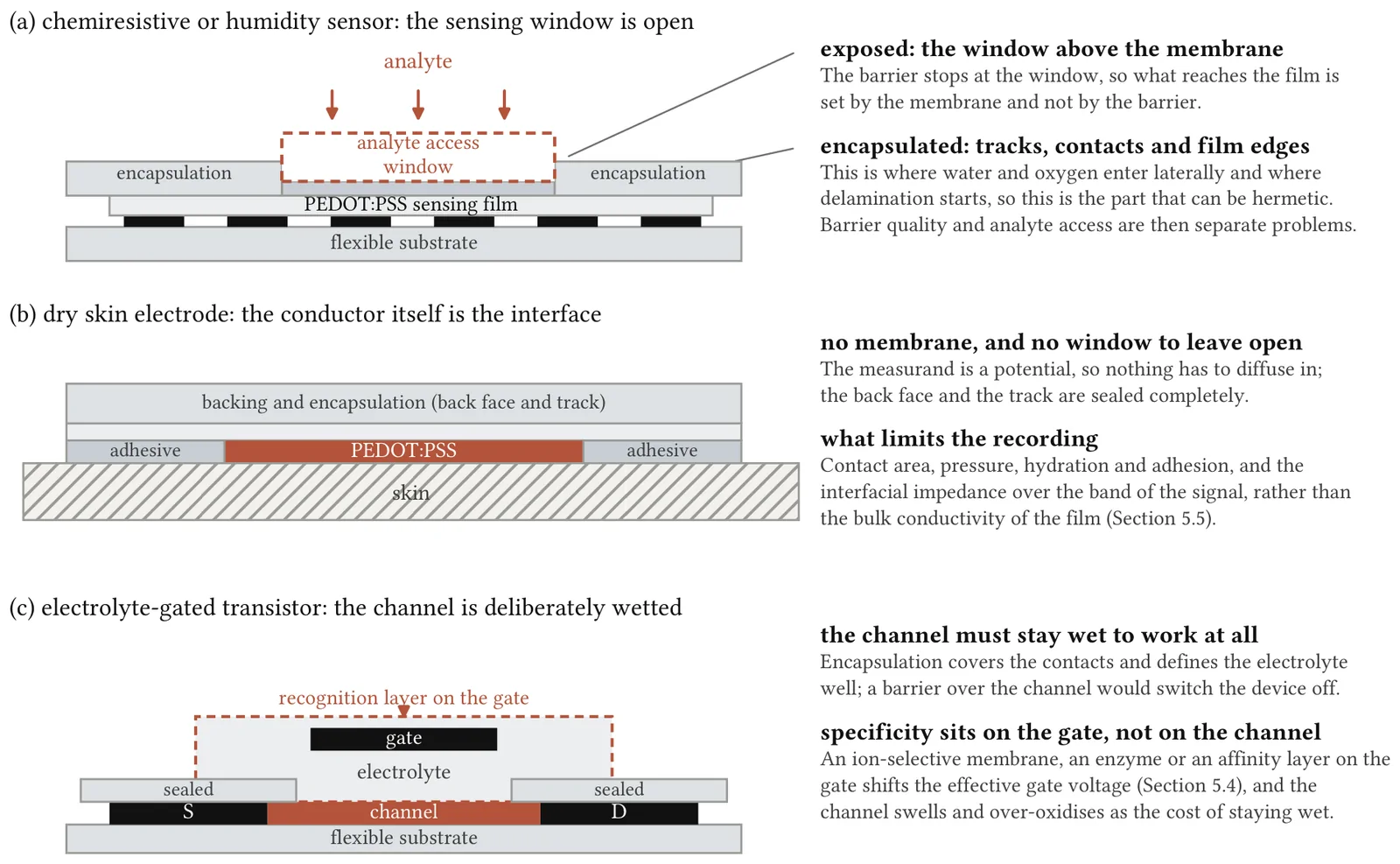
Representative cross-sections of three printed PEDOT:PSS device stacks.

Encapsulation decides how long the stack lasts. Multilayer barriers developed for flexible displays remain the reference solution against water and oxygen [82,83]. Applied to a sensor, however, a barrier cannot simply be laid over the device, and there is no single stack that serves every mode. The choice of what is sealed and what is left open is set by the measurand. Figure 3 draws in cross-section the three arrangements that cover most printed PEDOT:PSS devices. The first is for a chemiresistive or humidity sensor (Figure 3a), and the second is for a dry skin electrode (Figure 3b). The third is for an electrolyte-gated transistor, drawn here as an OECT (Figure 3c). Each panel is drawn to show which parts are sealed and which are deliberately left open. The panels are schematic and not to scale, and each shows one representative arrangement rather than a universal architecture. Electrode geometry and layer order also follow the application. For instance, the interdigitated geometry of Figure 3a is not used in Figure 3b,c. In addition, the layer order in Figure 3b is inverted with respect to the other two panels.

A chemical or humidity sensor cannot be hermetic, because the analyte must reach the film. The resolution is not a thinner barrier but a patterned one. The barrier covers the interconnects, the contacts, and the edges of the film, where lateral ingress and delamination begin. It stops at an opening above the sensing area, which is termed the analyte access window. What enters through that opening is then set by a selective membrane or by the porosity of the film itself, not by the barrier. Consequently, selectivity and barrier quality become separate design problems rather than one impossible problem. Stability for the part that remains exposed must come from reference channels or recalibration (Section 7).

An electrophysiological electrode inverts the problem. Its measurand is a potential, so nothing has to diffuse into the film, and the exposed face is the conductor itself. The back face, the track, and the contacts can be sealed completely. The design effort then moves to adhesion and to keeping the interface hydrated (Section 5.5).

An OECT inverts it again. Its channel must remain wetted to operate, so encapsulation covers only the contacts and the interconnects and serves to define the electrolyte well. Sealing the channel would switch the device off. Specificity sits on the gate, which carries the recognition layer, rather than on the channel (Section 5.4). In addition, the dominant failure modes are swelling, delamination, and over-oxidation of a layer that is permanently in contact with water (Section 6).

### 4.5. Print-to-Print Variation

Printed PEDOT:PSS films are defined by morphology rather than by lithographic geometry. Variation in drying, thickness, and annealing therefore propagates into baseline resistance and sensitivity [36,84]. Furthermore, roll-to-roll production adds web tension, register error, and drying gradients that a laboratory coater does not introduce [84,85]. In-line inspection by machine vision and terahertz spectroscopy is emerging as a response [86,87].

Print-to-print variation is also the main source of per-unit calibration effort, which Section 7.2 discusses. Hence, a process change that halves the spread in baseline resistance can matter more than one that doubles nominal sensitivity.

Manufacturing choices are usually reported separately from sensing results. Their sensing consequences are therefore made concrete below for four of the modes in Section 5. A thickness gradient across a printed sheet moves the baseline resistance of a piezoresistive device. Because the gauge factor depends on how close the network sits to percolation, the same gradient also disperses the sensitivity. The two errors are correlated and cannot be removed by a single scale factor. For a chemiresistor, drying rate sets the porosity that the analyte must enter. Consequently, two nominally identical prints with different drying histories differ in response magnitude and in response time, not only in baseline. For an OECT, the channel thickness enters the transconductance directly (Section 5.4). Hence, thickness scatter propagates linearly into device gain and must be absorbed by per-device calibration of the transfer curve. For an electrophysiological electrode, what matters is not the bulk conductivity but the hydrated volume that ions can reach and the area in contact with skin. Film thickness and the surfactant used for printability set the first, and surface roughness sets the second. In every case, the manufacturing variable that is easiest to control is not the one that dominates the sensing figure of merit. Therefore, reporting the batch structure (Section 8) is worth as much as reporting the mean performance.

In summary, the additives that make PEDOT:PSS printable also act as dopants. The measurand decides which parts of the device stack can be sealed. Finally, the printing process sets the unit-to-unit spread that calibration must absorb.

## 5. Transduction Modes of PEDOT:PSS Sensors

PEDOT:PSS transduces through several distinct mechanisms. Each mechanism exploits a different property of the film and has its own interferents, although water affects every mechanism. This section compares six groups of modes in turn: piezoresistive sensing, humidity and temperature sensing, chemiresistive gas sensing, OECTs, electrophysiological electrodes, and thermoelectric sensing. A final subsection compares their analytical performance, and Table 6 summarises the modes.

### 5.1. Piezoresistive Strain and Pressure

Strain changes the separation and contact between PEDOT-rich grains, and the film resistance follows. Latessa et al. [88] characterised the piezoresistive behaviour of flexible PEDOT:PSS films. Pristine films respond over a limited strain range before they crack. Most stretchable designs therefore use plasticised films, buckled films, or composites [38,57]. For instance, Seyedin et al. [75] made elastomeric, strain-responsive composite fibres of PEDOT:PSS in polyurethane.

Two penalties apply to such sensors. First, the polymer binder of most soft resistive sensors is viscoelastic, so the resistance depends on strain history as well as on strain [89]. Second, the film responds to humidity, so an uncompensated strain sensor partly measures water uptake (Section 6.1).

A film that survives a strain is not thereby a sensor for it. A strain sensor is specified by its working range, its gauge factor over that range, and its hysteresis. It is also specified by how completely the resistance recovers after unloading and by how all of these change after repeated cycling. Table 7 collects what the works cited in this review state in those terms. Every entry is a value stated by the cited work, and a dash means that the quantity was not reported there. The pattern of dashes in the upper block is itself a finding. In the PEDOT:PSS literature cited here, working range, gauge factor, hysteresis, and post-cycling behaviour are not reported together.

The PEDOT:PSS rows of the table record an absence rather than a ranking. Notably, the two studies with the most complete mechanical data characterise conductors and not sensors. Lipomi et al. [57] reported resistance against strain, crack density, and the effect of 1000 stretch cycles for a transparent electrode. Wang et al. [38] reported conductivity to fracture for a stretchable interconnect. Neither study states a gauge factor. The entry closest to sensing [75] separates reversible from irreversible deformation with a model of the filler network under tensile and cyclic loading. Even so, it relates the electromechanical response to that model rather than stating a gauge factor over a defined range. Piezoresistive characterisation of flexible PEDOT:PSS films exists [88]. What the field has not converged on is reporting range, gauge factor, hysteresis, and post-cycling recovery together. This lack of convergence is why Section 8 includes them in the proposed reporting set.

The two reference rows show what such reporting looks like. For a crack-based metallic-nanoparticle film, Aslanidis et al. [90] stated both quantities that the trade-off involves. They reported a strain sensitivity of $2.6\times 10^{8}$ at 7.2% strain over a working range of 0.1–7.2%. The authors describe this range as large. Compared with the elastomeric rows of the table, however, it is narrow, because the conduction path that gives the extreme sensitivity is close to broken. Section 9 treats this relation as the second trade-off. Similarly, Komolafe et al. [91] stated the durability of a screen-printed e-textile conductor in the terms that a garment needs. With domestic ironing as a repair step between cycles, the resistance change stayed below 20% after 400,000 bending cycles. It also stayed below 1 Ω after 50 wash cycles.

Moreover, two failure modes must be kept apart, because their remedies differ. First, cohesion can be lost inside the PEDOT:PSS layer. Within this layer, hydrogen bonds between grains are the dominant bonding mechanism, and water reacts with strained bonds to accelerate the separation [32]. Second, adhesion can be lost at an interface of the stack, as between the hole-transport and active layers of flexible organic solar cells [81]. Either mode ends the measurement. However, the first is addressed by plasticisation and crosslinking, and the second by adhesion layers and neutral-plane placement. Section 6.3 returns to the distinction.

### 5.2. Humidity and Temperature

Humidity and temperature are measurands in their own right and interferents for every other mode. Water uptake by the PSS phase changes inter-grain transport, which makes PEDOT:PSS responsive to humidity [31,92]. Temperature acts through thermally activated transport between grains. Wu et al. [69] and Kuzubasoglu et al. [47] used inkjet-printed PEDOT:PSS arrays and CNT/PEDOT:PSS films on textile, respectively, as flexible temperature sensors.

However, the two quantities are difficult to separate in a single film. A printed temperature sensor without humidity passivation measures both. Tachibana et al. [61] showed that bilayer passivation separates them.

### 5.3. Chemiresistive Gas Sensing

Redox-active gases change the doping level of PEDOT. Reducing gases such as ammonia de-dope a p-type conducting polymer, while oxidising gases such as nitrogen dioxide dope it further [93,94]. For instance, Seekaew et al. [48] detected ammonia with printed graphene–PEDOT:PSS films on flexible substrates. Similarly, Beniwal et al. [94] detected nitrogen dioxide at room temperature (RT) with flexible PEDOT:PSS films. RT operation removes the micro-hotplate of metal-oxide sensors and its power budget [95].

Selectivity is the structural weakness of this mode. PEDOT:PSS responds to many polar vapours and strongly to humidity. Therefore, selectivity is usually obtained at system level, through arrays of partially selective films and a classifier [96,97]. By contrast, Yang et al. [98] classified gases with a single printed In_2_O_3_ field-effect transistor, using bias-dependent features and machine learning.

### 5.4. Organic Electrochemical Transistors

The OECT uses mixed conduction as its operating principle. Under gate bias, cations from the electrolyte enter the PEDOT:PSS channel and de-dope its whole volume [99,100]. Because the volume participates, the steady-state transconductance scales with channel thickness, and it exceeds that of field-effect devices at comparable voltages [42,101]. Inal et al. [34] identified the product of mobility and volumetric capacitance as the governing material figure of merit.

The mechanism deserves a careful statement, because it is the only case in this review in which PEDOT:PSS provides gain rather than a passive response. In a resistive device, the analyte perturbs an existing conduction path, and the measured change is bounded by the fraction of the film that the analyte reaches. In an OECT, by contrast, the analyte acts on the gate. What reaches the channel is a shift in effective gate potential rather than the analyte itself. That shift de-dopes the full volume of the channel. Hence, the transduced charge is set by the volumetric capacitance $C^{*}$ of the polymer and not by its surface area. The transconductance follows as $g_{m}=\left(Wd/L\right)\;\mu C^{*}\left(V_{\mathrm{th}}-V_{g}\right)$, where *W*, *L*, and *d* are the channel width, length, and thickness, respectively [42,99]. Here, μ is the hole mobility, and $V_{\mathrm{th}}$ and $V_{g}$ are the threshold and gate voltages. Three consequences matter for sensing. First, the device can be made more sensitive by making the channel thicker, which has no analogue in field-effect sensing. Second, the governing material figure of merit is the product $\mu C^{*}$. This product couples an electronic and an ionic property and therefore cannot be maximised by ordering the polymer alone [34]. Third, the amplification occurs at the point of measurement, before any wiring. This local amplification is what allows micromolar-to-nanomolar limits of detection (LODs) in a thin-film device (Section 5.7).

However, the thickness term should not be read as a general design rule, because the expression holds under conditions that a sensor often violates. First, it assumes that cations penetrate the full thickness within the time of the measurement. Ionic transit through the channel is diffusive, so the time it takes grows faster than the thickness. The transconductance of an OECT is flat only up to a cut-off set by ion transport between the electrolyte and the channel. This cut-off is of the order of 1 kHz in the devices that established the millisiemens range [101]. For a measurand carried by a transient rather than by a steady level, bandwidth is the binding constraint, and a thicker channel trades gain for speed. Second, the expression assumes uniform de-doping, so the linear dependence on $\left(V_{\mathrm{th}}-V_{g}\right)$ describes a working window rather than the whole transfer curve. Third, the expression holds at a fixed W/L, electrolyte, and gate area, all of which change the result independently of the channel material [42,99]. The material figure of merit $\mu C^{*}$ is therefore defined at steady state. Its values should be compared across materials only together with the geometry and the bias at which they were obtained [34]. Thicker channels improve a steady-state measurement; they do not improve every measurement.

Sensing follows from any process that shifts the effective gate voltage. Ion concentration shifts it directly [102]. Enzyme-functionalised gates convert glucose concentration into a measurable current [103]. Gualandi et al. [104] and Coppedè et al. [105] used textile OECTs for wearable biosensing and ion-selective sweat monitoring, respectively. Bihar et al. [106] demonstrated a disposable paper OECT as an alcohol breathalyser. In addition, Hempel et al. [107] recorded cardiac electrophysiology with OECT arrays.

Stability limits this mode. Channels swell and delaminate in water unless they are crosslinked [41]. Furthermore, Kim et al. [20] showed that crystallinity and composition govern long-term aqueous stability. Bias stress and gate drift must be characterised before any stability claim is credible.

### 5.5. Electrophysiological Electrodes

Volumetric capacitance gives PEDOT:PSS a low impedance at skin and tissue interfaces [23,33], and that property is why the material entered bioelectronics. Khodagholy et al. [108] used conformal PEDOT:PSS arrays to record action potentials from the surface of the brain without penetrating it. The recordings remained stable beyond a week. On skin, PEDOT:PSS has been used in inkjet-printed tattoo electrodes, fully textile electrocardiogram (ECG) electrodes, and self-adhesive dry electrodes [70,109,110]. However, low material impedance is not by itself a statement about recording quality, and three distinctions separate the two. Table 8 collects the results in the terms those distinctions require. Its upper block is PEDOT:PSS. The lower block gives two reference systems, namely, a commercial dry-versus-wet comparison and a porous carbon stimulation electrode. The table supports no single ranking. A recording electrode is judged by impedance over the band of the signal, and a stimulation electrode by injectable charge and by endurance. The two material families are therefore strong in different columns.

The first distinction is that the interface takes three forms, not one. A hydrated interface, with an electrolyte or an ionic-liquid gel between conductor and tissue, is a different device from a dry electrode pressed against skin. Both differ from an electrolyte-gated channel, in which the polymer is the transistor rather than the contact. The first keeps the ionic network of the film swollen and so keeps the volumetric capacitance available. For instance, Leleux et al. [43] found that a gel applied to gold and to PEDOT:PSS electrodes held a low skin impedance steady over several days. The second must work with whatever hydration the skin supplies, which varies with site, time, and perspiration. Only one PEDOT:PSS study cited here, by Pani et al. [109], measured the same electrodes in both configurations under one protocol. In that study, textile PEDOT:PSS electrodes wetted with saline were stable and reproducible, and comparable to or better than disposable gelled Ag/AgCl electrodes. By contrast, the same electrodes used dry were more prone to noise artefacts during exercise, mainly because the skin contact was unstable. The third configuration is treated in Section 5.4. It appears here only to note that its figure of merit is transconductance rather than interfacial impedance. Results from the three are therefore not interchangeable.

The second distinction is that impedance is a spectrum, not a number. An electrode is useful if its impedance is low at the frequencies that the signal occupies. These frequencies differ by two orders of magnitude between an EMG and the slow components of an electroencephalogram (EEG). Hinrichs et al. [111] compared commercial dry and wet 19-channel EEG systems on the same subjects. They found no significant difference in alpha or beta power and comparable latency and amplitude for the P100 and P300 responses. Theta and delta power, in contrast, were higher with the dry electrodes. The two electrode types differed where the interfacial impedance is highest and the signal slowest, and agreed elsewhere. A single impedance value, usually quoted at 10 Hz or at 1 kHz, cannot express that difference. Reporting the spectrum over the band of interest is what makes two electrodes comparable.

The third distinction is that what limits a recording is often mechanical. Contact area, applied pressure, adhesion, and conformality decide how much of the nominal interface is in contact, and they change during movement. A thin-film system that achieves conformal contact removes the gap that produces motion artefact when a rigid sensor is held against skin by a band [112]. Similarly, Zhang et al. [110] reported a self-adhesive conducting-polymer dry electrode. In static and in dynamic measurement, its skin-contact impedance and noise were lower than those of both other dry electrodes and standard gel electrodes. Motion artefact, rather than impedance, then becomes the residual problem, but only under two conditions. The electrodes must be conformal and well adhered, and the recording must be short enough that the hydration state does not change. The claim does not hold over long recordings and on variable skin, because loss of hydration raises the impedance and loss of adhesion produces the artefact. The two failures progress together [43,109].

**Table 8:** Electrode results reported for PEDOT:PSS and two reference systems.

Electrode	Interface State	What Was Measured	Result as Stated
PEDOT:PSS			
Woven fabric treated with PEDOT:PSS [109]	Wet (saline)	Skin-contact impedance; ECG at rest and during exercise	Stable and reproducible; comparable to or better than disposable gelled Ag/AgCl
The same electrodes, used dry [109]	Dry	The same protocol	Operated without electrolyte; more prone to noise artefacts during exercise, mainly because of unstable skin contact
Inkjet-printed tattoo electrode, thickness <1 µm [70]	Dry, conformal	Contact impedance at 1 h and at 48 h against pregelled and dry references	Conformal adhesion from the sub-micrometre thickness; the film is perforable, so hair grows through it
Self-adhesive conducting-polymer dry electrode [110]	Dry, self-adhesive	Skin-contact impedance and noise, static and dynamic	Lower than other dry electrodes and than standard gel electrodes; ECG, EMG, and EEG on dry and wet skin and during movement
PEDOT:PSS and Au with an ionic-liquid gel [43]	Gel-assisted	Skin impedance over days	Low impedance held at a steady value over several days
Conformal array on the cortical surface [108]	In vivo, wet	Action potentials and local field potentials	Putative single units resolved without penetrating the surface; stable beyond one week
Reference systems			
Commercial dry versus wet 19-channel EEG [111]	Dry versus wet	Resting spectrum, P100, and P300 on the same subjects	Alpha and beta power not different (p>0.05); theta and delta higher with dry (p=0.0004 and p<0.0001). Evoked latency and amplitude comparable (p>0.10)
Three-dimensional micro- and nanoporous LIG, for stimulation [113]	Wet	Charge storage and injection capacity; endurance	362.4 mC cm^−2^ and 10.32 mC cm^−2^; storage capacity more than 100 times that of monolayer graphene and gold; >10^6^ stimulation cycles

Conducting polymers are not the only route to a low-impedance flexible electrode. The comparison with porous carbon electrodes is instructive, because the two families reach low impedance by different mechanisms. Porous carbon obtains its performance from electrochemically accessible surface area. For instance, Movaghgharnezhad et al. [113] wrote three-dimensional micro- and nanoporous graphene into a fluorinated polyimide film in a single laser step. The electrode reached charge storage and charge injection capacities of 362.4 mC cm^−2^ and 10.32 mC cm^−2^, respectively. The charge storage capacity is more than two orders of magnitude above that of monolayer graphene and gold electrodes. The electrode also endured more than a million stimulation cycles. The laser parameters are the levers that set that structure [46]. In contrast, PEDOT:PSS obtains its performance from a capacitance distributed through the volume of the film rather than across its surface [33,42]. The division of labour follows from the mechanism. Where charge must be injected into tissue repeatedly without faradaic damage, as in stimulation, accessible area and electrochemical robustness decide the outcome. Porous carbon is then the stronger candidate. Where the need is for a soft, conformal, low-impedance recording contact that can be printed from water onto a compliant substrate, the polymer has two advantages. These are its volumetric capacitance and its tissue-like modulus. Its limitations, discussed above, are its dependence on hydration and its swelling. Hybrid structures, in which a porous carbon scaffold carries a conformal polymer coating, follow directly from this division and are not yet characterised in common terms.

### 5.6. Thermoelectric Sensing and Harvesting

Doped PEDOT converts a temperature difference into a voltage through the Seebeck effect. Bubnova et al. [44] and Kim et al. [45] optimised the thermoelectric figure of merit of PEDOT-based films through control of the doping level. In particular, removal of excess dopant raised the RT figure of merit of PEDOT:PSS to a few tenths [45]. Free-standing PEDOT films and textile generators convert body heat into power and into a temperature signal [25,114,115]. However, the output voltage is small, so the readout must be low in noise and the doping level must remain stable.

### 5.7. Analytical Performance: Sensitivity, Detection Limit, Linearity, and Interference

The modes above are usually compared by response magnitude. For chemical and biochemical sensing, however, the relevant figures of merit are different. They are the LOD, the linear range, the selectivity against a defined interference panel, and the response and recovery times. Table 9 collects the values that the studies cited in this review state explicitly, together with representative results obtained with other material classes, for context. Every entry is taken from the explicit statement of the cited work. A dash means that the quantity was not stated there, and “LOD not stated” marks a range reported without an LOD. Both gaps are findings discussed below.

Two conclusions follow, and they point in opposite directions.

The first conclusion is that, as a bare chemiresistor, PEDOT:PSS is not competitive on LOD. For instance, Beniwal et al. [94] resolved nitrogen dioxide over 0.5–50 ppm at RT with a drop-cast PEDOT:PSS film on screen-printed graphene–carbon electrodes on paper. The response was 0.79–12.97% across that range. Other material classes reach the parts-per-billion decade. Abegg et al. [116] detected nitrogen dioxide down to 3 ppb with a heated WO_3_ chemiresistor at 50% RH. Kim et al. [117] reported LODs of 50–100 ppb for volatile organic compounds with Ti_3_C_2_T_x_ MXene chemiresistors at RT. Moreover, a PANI composite with inorganic sensitisers gave about 4.4 times the response of plain PANI at 1 ppm ammonia [118]. Section 5.4 gives the physical reason. A chemiresistor converts analyte adsorption into a small perturbation of an already-conducting network, and nothing in that chain provides gain.

**Table 9:** Analytical performance of PEDOT:PSS sensors and of other material classes.

Material and Format	Analyte	Range and LOD	Reported Performance and Conditions
PEDOT:PSS			
Drop-cast film on screen-printed graphene–carbon interdigitated electrodes, paper [94]	NO_2_	0.5–50 ppm; LOD not stated	Response 0.79–12.97% over the range; response and recovery 210 s and 60 s at 0.5 ppm; RT (27 ± 2 °C)
Inkjet-printed graphene–PEDOT:PSS film [48]	NH_3_	–	RT operation on a flexible substrate
OECT, Pt-nanoparticle and glucose-oxidase gate [103]	Glucose	LOD ≈ 5 nM	Three orders of magnitude below the same device without gate nanomaterials
Textile OECT with ion-selective membranes [105]	Sweat electrolytes	10^−5^–1 M; LOD not stated	Selectivity assessed against interfering ions; membrane chosen by ionophore
Paper OECT with alcohol dehydrogenase [106]	Ethanol vapour	Breath ethanol equivalent to 0.01–0.2% blood alcohol concentration; LOD not stated	Disposable breathalyser; enzymatic reaction lowers the drain current
Fully textile OECT [104]	Adrenaline, dopamine, and ascorbic acid	Adrenaline, linear in log concentration over $3\times 10^{-5}$–$5\times 10^{-4}$ M; LOD $1\times 10^{-5}$ M	Real-time detection both immersed and in wearable operation
Other material classes, for context			
PANI with N-doped graphene quantum dots on hollow In_2_O_3_ nanofibres [118]	NH_3_	0.6–2.0 ppm; LOD not stated	Response 15.2 at 1 ppm, about 4.4 times that of plain PANI; RT
Flexible chemiresistive glucose-oxidase biosensor on LIG electrodes [50]	Glucose	0.01–3.0 mM; LOD 0.8 µM	Selectivity reported; LOD at a signal-to-noise ratio of 3
LIG enzymatic electrode [49]	Glucose	Up to 8 mM; LOD 0.431 mM	Amperometric, 43.15 µA mM^−1^ cm^−2^; no interference from ascorbic acid, uric acid, or urea; flexible polyimide substrate

The second conclusion is that, as a transistor channel, PEDOT:PSS changes category. The same material in an OECT with a functionalised gate detects glucose with a limit of approximately 5 nM [103]. This limit is three orders of magnitude below that of the same device without gate nanomaterials. The gain is local and electrochemical rather than electronic, and it is available precisely because the polymer conducts ions as well as holes. Flexible enzymatic glucose sensors on LIG reach LODs of 0.8 µM [50] and 0.431 mM [49]. For the same analyte, the OECT route therefore reaches LODs lower by orders of magnitude.

Taken together, the two conclusions support a balanced statement about the material. PEDOT:PSS is not the most sensitive sensing layer available. Rather, it is the one that can be printed from water onto a soft substrate and operated at RT without a heater. It can also be configured either as a passive resistor or as a locally amplifying transistor using the same ink. Where an application needs the lowest possible LOD and can tolerate a hotplate, a metal oxide is the better choice. Where it needs conformal contact, RT operation, and a recognition layer, the OECT configuration is competitive on analytical terms and unmatched on form factor.

**Figure 4:**
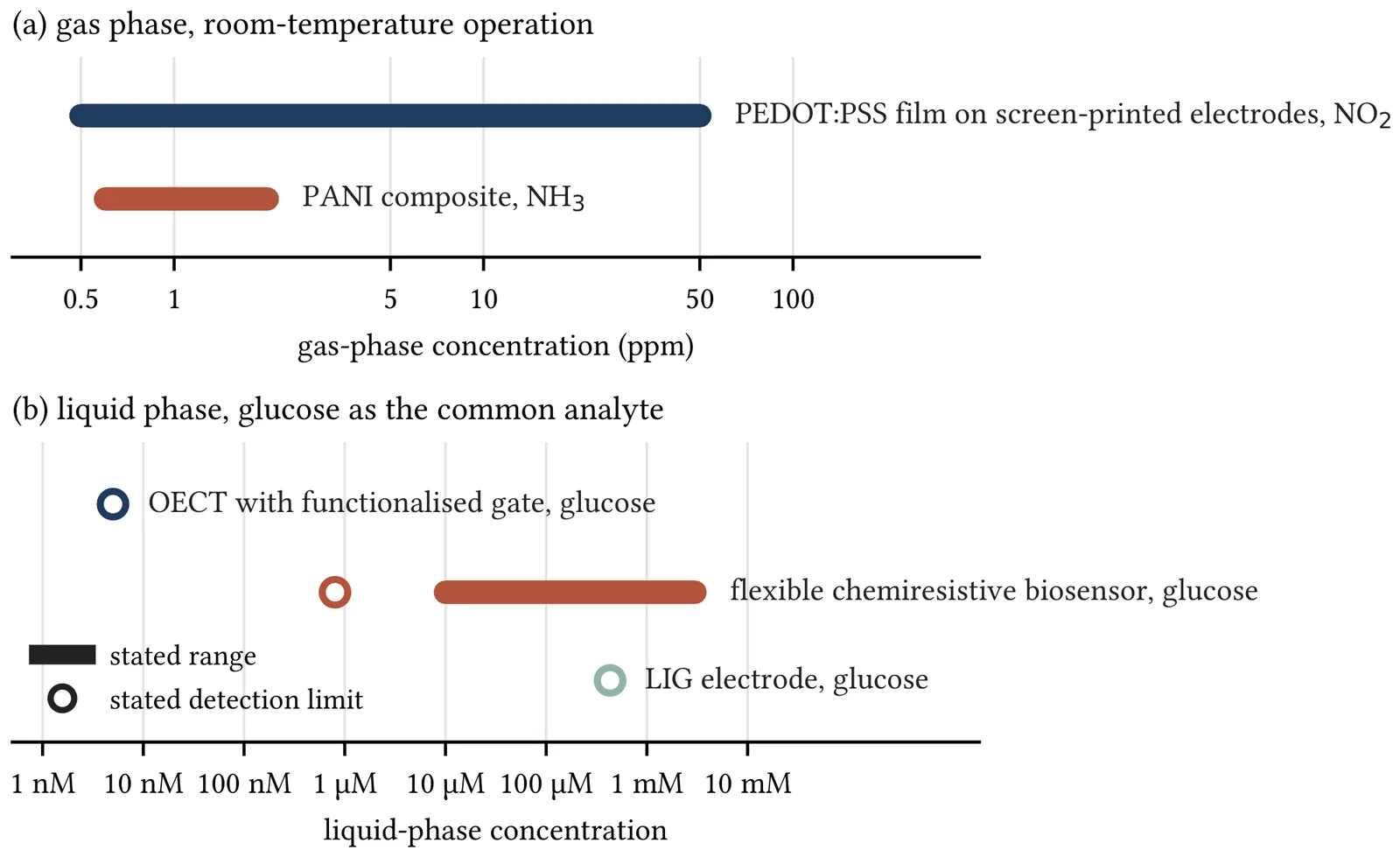
Reported working ranges and LODs: (**a**) gas phase; (**b**) liquid phase.

Figure 4 places these values on concentration axes. It plots the gas-phase rows that state a range in parts per million and the glucose rows of Table 9 that state a range or a limit. The sweat-ion and catecholamine entries concern other liquid-phase analytes. The breath-ethanol range is stated as a blood alcohol equivalent rather than a gas concentration. None of these three entries is plotted. The gas phase and the liquid phase are shown separately, because the two cannot share a scale. Figure 4a covers the gas phase at RT. It shows a PEDOT:PSS film over 0.5–50 ppm NO_2_ [94] and a PANI composite over 0.6–2.0 ppm NH_3_ [118]. Figure 4b covers the liquid phase, with glucose as the common analyte. It shows an OECT with a functionalised gate at an LOD of about 5 nM [103]. It also shows a flexible chemiresistive biosensor over 0.01–3.0 mM with a limit of 0.8 µM [50]. The third entry is an LIG electrode with an LOD of 0.431 mM [49]. Its linear range is stated only as extending up to 8 mM, so it carries no bar. Open circles mark a stated LOD, and bars mark a stated range. The entries are examples drawn from the works cited in this review, not a survey of best reported values. The contrast between the two PEDOT:PSS entries is the point of the figure. As a bare resistor, the polymer works in the parts-per-million decade of a gas. As a transistor channel with a functionalised gate, it reaches an LOD of about 5 nM in solution.

Assembling Table 9 was itself informative. Among the PEDOT:PSS sensing studies surveyed here, three items were rarely available together. These were a stated LOD, a linear range, and an interference panel measured at a stated RH. The most common omission is humidity. The baseline of this material is set by water uptake (Section 6.1). Hence, an LOD quoted without the humidity at which it was obtained is not transferable to another laboratory. The second most common omission is the interference panel. For chemiresistive PEDOT:PSS sensors, selectivity is usually obtained computationally rather than physically (Section 9). Therefore, the list of interferents against which a device was tested is the only evidence that the computational route is sound. Humidity is a core item of the reporting set in Section 8. In addition, Section 10 identifies an agreed interference panel at a stated humidity as the step that would make computational selectivity auditable.

In summary, every transduction mode of PEDOT:PSS draws on the same morphology, and every mode inherits its sensitivity to water. As a bare chemiresistor, the polymer works in the parts-per-million decade, whereas an OECT with a functionalised gate reaches nanomolar limits. Electrodes are judged by impedance over the signal band and by contact stability, not by material impedance alone. Section 6 treats the sensitivity to water together with the other routes to drift.

## 6. Stability, Drift, and Lifetime

A printed PEDOT:PSS sensor is a hygroscopic, chemically active, and incompletely encapsulated film that must hold a calibration. However, most reports characterise devices within days of fabrication, whereas deployments last months. This section treats the resulting drift in five parts. The first four subsections examine four routes to drift, namely, humidity, ageing, mechanical fatigue, and washing. The fifth subsection states which mechanism dominates in which sensor class and what each mechanism implies for correction.

### 6.1. Humidity Uptake

Water is the main interferent for PEDOT:PSS. Water uptake by PSS changes inter-grain transport and softens the film [31,35]. For instance, Kawano et al. [31] traced the degradation of organic solar cells in air to water absorbed by the PEDOT:PSS layer. Moreover, Dupont et al. [32] reported that moisture accelerates the decohesion of PEDOT:PSS films under mechanical stress.

Therefore, for sensing, both the baseline and the sensitivity depend on RH. The size of this dependence varies with treatment, because treatments change the amount and distribution of PSS [19,27]. Consequently, the RH during every measurement is a minimum reporting item. In addition, a reference element exposed to humidity but shielded from the measurand allows the humidity response to be compensated.

### 6.2. Thermal, Oxidative, and Electrochemical Ageing

PEDOT:PSS loses conductivity under thermal stress through changes in the PSS phase and in the grain contacts [36,119]. In addition, electrochemical operation adds over-oxidation and de-doping, which matter most for transistors and electrodes [20,23]. The electronic-nose literature shows that chemical-sensor drift is smooth in time and largely common-mode across an array. It also shows that, left uncorrected, this drift is severe enough to erase classifier accuracy within months [26]. Notably, that description was established on metal-oxide gas-sensor arrays under controlled exposure. It applies to the ageing treated here and to the humidity response of Section 6.1, and Section 6.5 states where it stops applying. Because ageing drift accumulates smoothly in time, it should be reported as a time series of baseline and sensitivity, not as a single retention percentage.

### 6.3. Mechanical Fatigue and Delamination

Cyclic bending shortens the life of flexible thin-film devices sharply once the applied strain exceeds a critical value [80]. In flexible organic solar cells, weak adhesion between layers was the main cause of such loss [81]. Cohesive failure inside the film and adhesive failure at an interface should therefore be reported separately, because their remedies differ. Cohesive failure occurs inside the PEDOT:PSS layer, in which hydrogen bonds between grains are the predominant bonding mechanism. Water from the environment reacts with strained hydrogen bonds, so the decohesion rate depends on moisture content, temperature, and applied stress [32]. In contrast, adhesive failure occurs at an interface, either between the film and its substrate or between two layers of the stack. The number of bending cycles before such failure depends on the adhesion across that interface [81]. Cohesive failure is addressed by plasticisation, crosslinking, and limiting the working strain below crack onset (Section 5). Adhesive failure is addressed by adhesion layers and by placing the film near the neutral plane. Notably, moisture accelerates cohesive failure [32], which is why mechanical and environmental ageing cannot be qualified independently for this material.

Furthermore, a viscoelastic polymer binder makes the resistance of a conductive composite time-dependent, and this resistive relaxation is a deterministic function of load history [89]. Hence, a memoryless calibration curve is the wrong model class for such sensors. A fatigue report should state the bending radius, the cycle number, the strain state, and the measured quantity after cycling.

### 6.4. Washing and Wear

Textile PEDOT:PSS sensors face laundering, which combines agitation, heat, surfactants, and repeated wetting. Reported survival depends strongly on the washing protocol, and standardisation has been argued for [120,121]. Notably, encapsulation and interconnect design usually decide the outcome more than the sensing film does [91].

### 6.5. Which Mechanism Dominates in Which Sensor Class

The preceding subsections describe separate mechanisms. It is worth stating explicitly that these mechanisms do not produce the same drift and do not yield to the same correction. A reversible humidity response, irreversible conductivity loss through ageing, electrochemical de-doping under bias, a crack, a delamination, and a change of skin contact differ in time constant. They also differ in whether they recover and in whether a second element placed beside the first would experience them identically. Table 10 maps each mechanism to the sensor classes in which it dominates, its signature in the signal, and its reported mitigation. Its Reversible and Common-Mode columns decide the correction strategy. Only a mechanism that is smooth and shared between a sensing element and a reference element placed beside it can be removed by common-mode subtraction.

Two consequences follow. The first concerns water. Humidity reaches every mode, which is why it is treated first. However, it is also the most tractable term. It is reversible, it is shared between a sensing element and a reference element in the same package, and it can be bounded by passivation. Calling it the dominant problem is accurate for a device characterised over hours but misleading for one deployed over months. In such a deployment, the irreversible mechanisms in Table 10, ageing among them, set the service life.

The second consequence concerns the correction methods of Section 7. The first two rows of Table 10 are smooth and largely common-mode, which is the regime in which the electronic-nose drift literature was developed [26]. In contrast, the remaining rows are not. A crack is a step, a delamination is an open circuit, and an electrode that dries out affects one channel and not its neighbour. Consequently, methods built on the smooth, common-mode assumption transfer to chemiresistive arrays and to slow ageing. However, they should not be assumed to transfer to strain sensors, to biased transistors, or to skin electrodes without evidence that the assumption holds there.

In summary, humidity, ageing, fatigue, and washing all move the baseline and the sensitivity of a PEDOT:PSS sensor. However, they do not move them in the same way, and they do not yield to the same remedy. Materials design cannot remove these changes entirely, so Section 7 treats the compensation of what remains.

## 7. From Raw Signal to Calibrated Measurement

A PEDOT:PSS sensor rarely produces a directly usable quantity. Its resistance, current, or voltage depends on the measurand and also on humidity, temperature, and history. This section treats the calibration layer in three parts: the front end, the compensation of drift and unit-to-unit variation, and the role of uncertainty.

**Figure 5:**
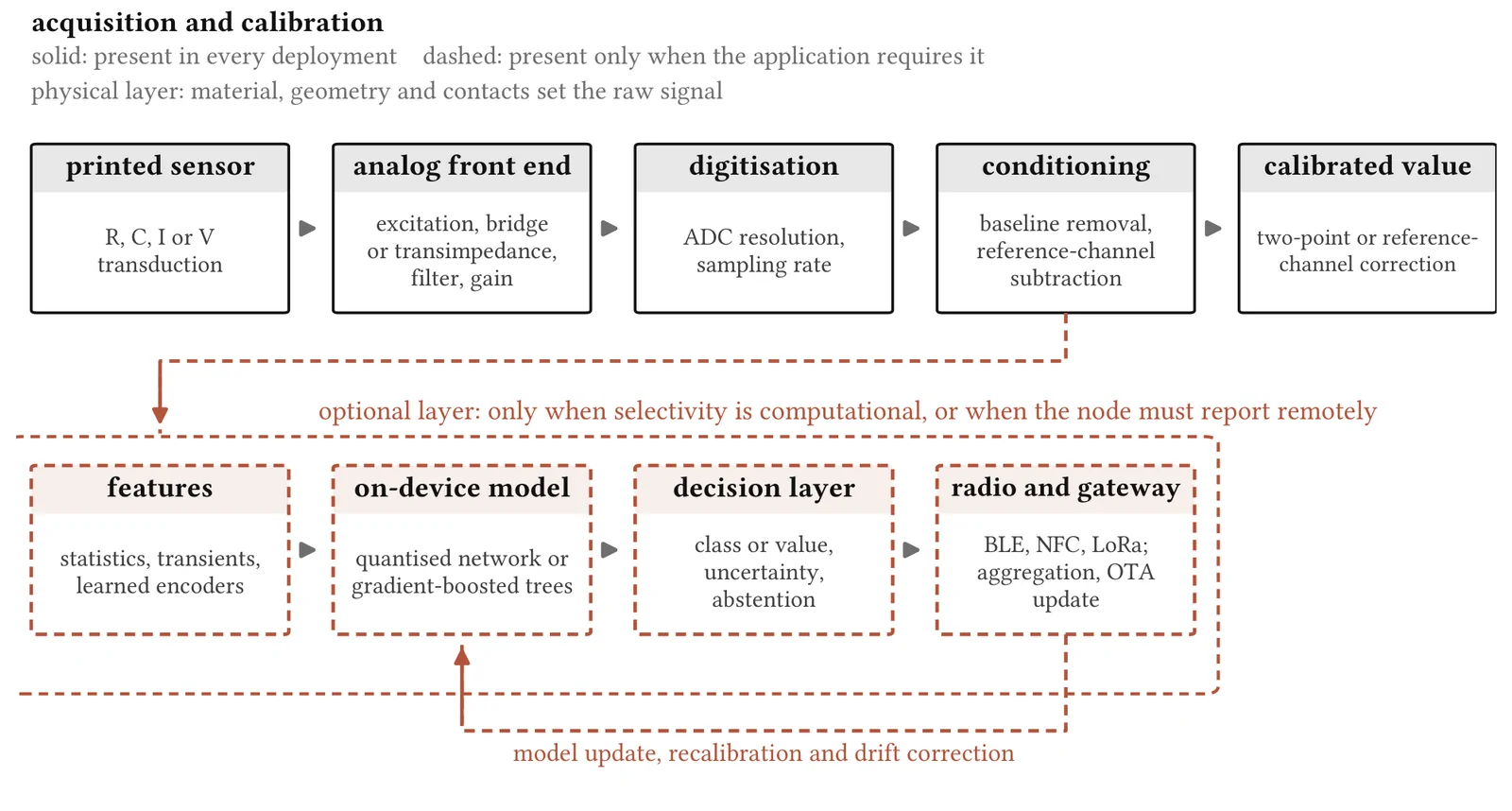
Signal chain of a printed PEDOT:PSS sensor node.

Figure 5 shows the signal chain. In the figure, ADC denotes the analogue-to-digital converter, BLE Bluetooth Low Energy, NFC near-field communication, LoRa long range (a low-power wide-area radio), and OTA an over-the-air update. The solid blocks are present in every deployment. The physical layer sets the raw signal, and the analogue front end and digitisation set what is recoverable. Conditioning plus a two-point or reference-channel correction already yields a calibrated value. The dashed blocks in the lower lane are optional. They are needed only where selectivity must be obtained computationally (Section 9) or where the node must report without a wired instrument beside it. The dashed return path is the recalibration loop, and its cost is set by the stability achieved in Section 3 and Section 6.

Not every PEDOT:PSS sensor needs the whole chain, and the top row of Figure 5 is a complete instrument on its own. The top row suffices when three conditions hold. First, the measurand is physical rather than chemical. Second, the device is read at a known temperature and humidity. Third, the deployment is short enough that ageing has not moved the baseline. Under these conditions, a two-point calibration against a reference instrument, together with a reference element for common-mode subtraction, is sufficient. Nothing in the lower lane is then required. In contrast, the lower lane becomes necessary in the two situations named on its label in Figure 5. The first arises when selectivity has to be recovered from the pattern across an array rather than from a membrane (Section 9). The second arises when the node must report remotely, so that readings and recalibration updates pass over the radio link. Within that lane, the abstention rule of the decision layer is justified when a confident wrong reading would have severe consequences (Section 7.3). Treating the lower-lane blocks as compulsory inflates the cost of a measurement that a bridge and a two-point fit would have delivered.

### 7.1. Front End and Reference Channels

The front end must match the transduction mode. Resistive films need a ratiometric bridge with a matched dummy element, whereas OECTs need a stable gate bias. Electrophysiological electrodes need a high-impedance instrumentation amplifier [122,123,124].

A reference channel is the most effective single addition for a PEDOT:PSS sensor. An identical film, shielded from the measurand but exposed to humidity and temperature, converts the main interferents into a subtraction. In addition, sampling should preserve the transient shape of the response, since that shape often carries information that the steady-state value lacks [125]. Tasdemir et al. [126] showed this with a single metal-oxide gas sensor. Under product-grouped cross-validation, a raw-waveform network graded teas with a macro-F1 of 0.811, against 0.624 from summary statistics. Likewise, in laser welding, a single optical microphone sampled at 2.5 MHz was shown to be a viable alternative to multi-sensor stacks [127]. From that one signal, a compact convolutional network classified five weld states with a macro-F1 of 0.72 under replicate-out cross-validation.

### 7.2. Drift Compensation and Calibration Transfer

Three problems are commonly conflated. Drift is the change of one device over time. Unit-to-unit variation is the difference between nominally identical printed devices. Domain shift is the difference between calibration conditions and conditions of use.

Drift that is smooth and largely common-mode responds to component correction and online adaptation [26,128,129]. Water uptake and slow ageing produce drift of this kind, whereas the step-like mechanisms in the lower rows of Table 10 do not. For instance, Dong et al. [128] reported one such adaptive framework, which extended the useful life of a gas-sensor array by four to ten months. Its model-adjustment time was below 300 ms. Where the mechanism is a crack, a delamination, or an electrode losing contact, the same methods will absorb the step into the correction. They then return a plausible but wrong value, which motivates the abstention rule of Section 7.3.

Unit-to-unit variation, by contrast, is best reduced in the process (Section 4). Where it remains, calibration transfer maps a new unit onto a master calibration from a few paired measurements [130,131]. Finally, domain shift is addressed by domain adaptation without labels in the target condition [132,133].

The criterion for choosing a method is the number of labelled measurements that a deployment can afford per unit and per recalibration interval. Hence, a method that recovers accuracy with five paired samples is more useful than one that needs fifty. Field experience with low-cost air-quality sensors also shows that calibration must be maintained, not performed once [134,135]. In addition, compact correction models can run on the microcontroller beside the sensor [136]. Heavier models can instead be partitioned between the device and a nearby edge server [137]. The energy cost of such inference depends on the accelerator and on its software stack [138].

### 7.3. Uncertainty and Abstention

A deployed PEDOT:PSS sensor will meet conditions outside its calibration set, such as a delaminated contact or a dried electrode. A model that always returns a confident value will then report a plausible but wrong number. Conformal prediction provides distribution-free coverage under exchangeability and adds negligible cost at inference [139,140]. This guarantee is not assured under such conditions, so conformal prediction must be paired with a drift or out-of-distribution check. In addition, a reject option formalises the decision to abstain [141]. In drug-discovery triage, an uncertainty and applicability-domain gate acted as an abstention mechanism rather than an accuracy gain, and this abstention reduced coverage [142]. A sensor model faces the same trade-off, because every abstention leaves a measurement unreported. Notably, for chemiresistive arrays with computational selectivity, abstention distinguishes a usable instrument from an unfalsifiable one.

In summary, reference channels, drift correction, calibration transfer, and abstention form the calibration layer from which each PEDOT:PSS sensor draws what its deployment requires. Their cost depends directly on the stability and uniformity achieved in Section 3, Section 4, Section 5 and Section 6.

## 8. A Minimum Reporting Set for PEDOT:PSS Sensors

Comparing PEDOT:PSS sensors across studies is difficult, because key conditions are often missing. This section addresses that gap in three parts. The first subsection states the problem. The second lists a minimum reporting set and the criteria by which its items were chosen. The third explains two items in detail.

### 8.1. The Problem

Studies of nominally similar PEDOT:PSS sensors frequently omit the information needed to compare them. For instance, a conductivity is quoted without the commercial grade, secondary dopant, or annealing history. A sensitivity is quoted without the RH at which it was measured. A classification accuracy is quoted without stating whether the test data came from the device used for training.

The sensor community has raised such gaps in general terms [143]. Similarly, the e-textile community has raised them for washing [120], and materials informatics has raised them for data and code [144,145].

**Figure 6:**
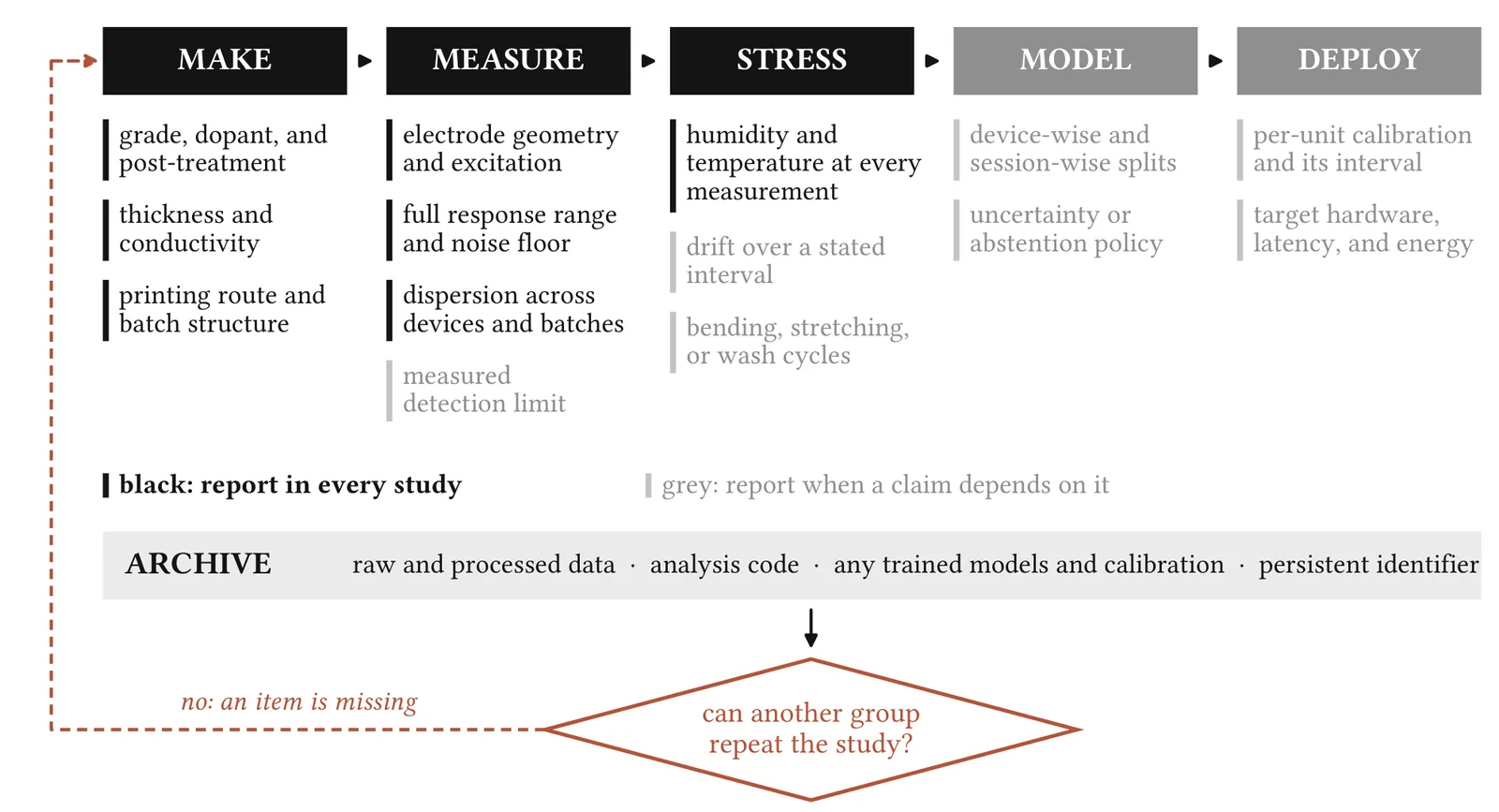
Structure of the proposed minimum reporting set.

Figure 6 shows the structure of the proposed set, and Table 11 lists its items. The figure follows the order in which a study proceeds. Its six steps group the stages of Table 11. Make covers Material and Process, and Measure covers Device, Metrology, and Statistics. Stress covers Environment and Reliability. Model and Deploy correspond to the Model and Deployment stages, and Archive covers Artefacts. The figure shows the shape of the set rather than its contents. Under each step, the black entries are the items to report in every study. The grey entries are those to report when a claim depends on them. The Model and Deploy steps are grey as a whole, because they apply only to studies with a trained model or a deployment claim. For each item, Table 11 also gives the use that the item enables. Its Scope column separates the items that apply to every study (Core) from those that apply when a claim depends on them (Conditional). Only the conditional items may require an experiment that the study would not otherwise have performed. The closing test of the figure states the purpose of the whole set. If another group cannot repeat the study, an item is missing.

### 8.2. The Items

Table 11 lists the set. In its Device row, DC and AC denote direct and alternating current. The set is deliberately short, and the items were not chosen by listing everything that could be measured. Three criteria were applied, and an item is included only if it meets all three.

The first criterion is that an item changes how a reported number must be read. A conductivity without the secondary dopant, or a sensitivity without the RH, is not a weaker result. Rather, its value cannot be placed on the same axis as the values reported by other studies. Items that merely add detail without changing interpretation were left out.

The second criterion is that an item costs reporting rather than work, with three named exceptions. Most items are already known to the group that performed the study. For instance, the anneal temperature was set, the humidity was that of the laboratory, and the number of devices was counted. However, three items carry an experimental cost, and claiming otherwise would overstate the case. First, a measured LOD requires replicate measurements at measurand levels near the limit, which not every study performs. Second, a drift figure requires the device to be held and re-read over a stated interval. Third, a washing or cycling protocol is an experiment in its own right. These items are marked conditional in Table 11 and are asked only of studies whose claims rest on them. Accordingly, a disposable strip needs no washing protocol, and a paper that makes no LOD claim need not measure one. Where an item would have required new work in every case, such as an accelerated ageing model, it was excluded. Instead, it was left to Section 10 as an open problem.

The third criterion is that the absence of an item blocks a specific downstream use. The right-hand column of Table 11 names that use in each case. If no concrete use could be named, the item was dropped.

Regarding practical applicability, the set is intended to be used at three points. First, an author can run it as a final check before submission, in the order of the table. That order follows the order in which the information is generated. Second, a reviewer can use the right-hand column to state precisely what a missing item prevents, rather than asking for more detail in general. Third, a reader performing a meta-analysis can use it as an inclusion filter, since a study reporting the Material, Process, and Environment rows is comparable with others. A study that does not report them is effectively a single-laboratory result. The Model row applies only to studies that include a trained model, and the Deployment row only to studies that make a deployment claim. The Reliability row applies in proportion to the claimed application.

### 8.3. Two Items That Deserve Emphasis

The first item is humidity during measurement. PEDOT:PSS responds to water in every mode described in Section 5. Consequently, a sensitivity or drift figure without the RH at which it was measured cannot be compared with any other. Reporting humidity costs one sensor and one column in a table.

The second item is device-wise evaluation. A model trained and tested on data from one physical sensor measures how well it has learned that sensor. For printed devices, where unit-to-unit variation dominates, a sound evaluation trains on some devices and tests on others. The difference between the two results estimates the calibration-transfer burden. Random splitting of correlated samples inflates the result. For a metal-oxide electronic nose, macro-F1 was 0.68 under leave-one-cut-out validation and reached 0.97 when samples were split at random [146]. The same inflation arises wherever records repeat. In a food antioxidant dataset in which 39.4% of records shared a product name, a random split rewarded memorisation [147]. In addition, the device-wise result should be reported as the mean and spread across devices and batches, not as the result of the best device alone.

In summary, this section has proposed a minimum reporting set for printed PEDOT:PSS sensors. Each item changes how a reported number must be read, and its absence blocks a specific downstream use. Most items cost reporting rather than work, and the three items that require an experiment are asked only of studies whose claims rest on them. Humidity during measurement and device-wise evaluation deserve particular emphasis. The set is proposed as a checklist, not as a standard. A standard requires community adoption that no single review can confer.

## 9. Discussion: Three Trade-Offs and What the Literature Has Settled

The preceding sections are organised by topic. This section confronts their main findings with one another, because the difficulties of PEDOT:PSS sensing are not independent problems but three recurring trade-offs seen from different angles. For each trade-off, this review states what the literature has settled, what remains contested, and what is simply absent. The section proceeds in four parts. The first three parts treat conductivity against baseline stability, sensitivity against linearity and repeatability, and physical against computational selectivity. The fourth part draws the practical consequences for a reader choosing a route.

### 9.1. Conductivity Against Baseline Stability

What is settled is that every route to high conductivity acts on the same phase. DMSO, ethylene glycol, polyols, acids, alcohols, and ionic additives all remove, rearrange, or screen PSS [21,22,54,55]. The mechanism is no longer in dispute. These treatments change the morphology and the doping level, but not the polymer itself [16,18,30].

The open question is whether the aggressive removal of PSS improves or degrades stability. Removing PSS removes hygroscopic material, which should reduce humidity sensitivity. However, the same treatment plasticises or thins the film and opens it to oxygen and water [19,32]. Reports exist on both sides, and they are difficult to reconcile because the humidity during measurement is rarely stated.

The literature lacks a humidity coefficient reported alongside conductivity. This is the single most consequential gap identified in this review. The field has an agreed figure of merit for the property it optimises, but no agreed figure of merit for the property that limits deployment. Therefore, Section 8 makes RH a core reporting item rather than a conditional one.

### 9.2. Sensitivity Against Linearity and Repeatability

What is settled is that, in crack-engineered films, sensitivity comes from the fragility of the conducting network and rises as the network approaches disconnection [90]. In PEDOT:PSS films, the same mechanism is consistent with the reported piezoresistive data [57,88], although Table 2 marks the link as inferred. This limits the combination of sensitivity and linearity in any single film, since a device cannot be both at the percolation edge and far from it.

It is unresolved whether the high gauge factors reported for crack-engineered or heavily plasticised films survive repeated cycling. Hysteresis and creep are deterministic consequences of the viscoelastic matrix [89], not noise, and they are reported inconsistently.

The missing quantity is the dispersion across devices. Sensitivity is usually quoted for the best specimen. For a printed, morphology-defined film, that value describes the tail of a distribution rather than the process.

### 9.3. Physical Selectivity Against Computational Selectivity

What is settled is that PEDOT:PSS has low intrinsic selectivity. It responds to redox activity, to polarity, and strongly to water. No treatment in Section 3 changes that in kind. The field has largely accepted that selectivity is obtained at the system level. The recognised sources are partially selective arrays and a classifier [96,97], or a membrane or recognition layer placed in front of the film [102,103].

The debate concerns how far the computational route can be pushed before it becomes unfalsifiable. A model that maps any pattern to a class will also map an unseen interferent to a class. A literature on abstention and calibrated uncertainty exists [139,141]. However, it has barely been applied to printed chemical sensors.

The missing element is the interference panel. Very few PEDOT:PSS gas-sensing reports state the response to a defined set of interferents at a defined humidity. Such reporting is what would make the computational route auditable.

### 9.4. What This Means for a Reader Choosing a Route

Three practical consequences follow. First, a device whose output is a magnitude at a single time point, such as a chemiresistor, inherits all three trade-offs. It also needs the full calibration layer of Section 7. Second, a device whose output is a change on a short timescale, such as an electrophysiological electrode, is largely insulated from the slow drift terms. It is not insulated from the loss of hydration and adhesion that develops over long recordings (Section 5.5). For short recordings, it is correspondingly closer to deployment. Third, a device whose output is a ratio against a reference element removes the common-mode baseline drift behind the first trade-off. The reference element costs one extra printed film, and this ratiometric design is the least expensive reliability improvement available in this material system.

In summary, the three trade-offs share one structure, with a settled basis, a contested question, and an absent measurement. The absent measurements are a humidity coefficient reported alongside conductivity, the dispersion across devices, and an interference panel at a defined humidity. The type of output, whether a magnitude, a change, or a ratio, indicates how far a device inherits these trade-offs.

## 10. Open Problems and Outlook

This section states seven open problems that, in the authors’ view, decide whether printed PEDOT:PSS sensors move from demonstration to deployment. It then states four limitations of this review.

Stability is the bottleneck, not conductivity. Conductivities above $10^{3}$ S cm^−1^ are available from several treatments [28,29,54]. However, applications lack a baseline that remains stable under changing humidity. Hence, the highest-value work lies in crosslinkers that do not raise impedance, encapsulation compatible with analyte access, and quantitative ageing models.

Treatments should be selected for the measurand. The treatment that maximises conductivity is seldom the one that minimises humidity sensitivity or drift. Reporting a humidity coefficient alongside conductivity would make this trade-off visible.

Humidity cross-sensitivity needs a standard test. Rotzler et al. [120] showed, for e-textile washing protocols, how a missing standard blocks synthesis across studies. A common humidity-cycling protocol for PEDOT:PSS sensors would close a similar gap.

Formulation is under-explored by data-driven design. Machine learning has been applied widely to polymer property prediction from molecular structure [145,148,149]. However, it has been applied much less to formulation and process variables, such as additive fraction, annealing profile, and printing settings [150]. Within PEDOT:PSS, these variables change performance more than the choice of grade does.

Calibration cost per unit should become a reported figure of merit. A sensor whose per-unit calibration cost does not fall with volume cannot scale. The open question is how few paired measurements per unit suffice. A further question is whether process control can remove the need for such measurements entirely.

Selectivity needs a route that can be audited. This is the central open problem for chemical sensing with PEDOT:PSS, and it has four candidate routes, none of them yet closed. First, selective membranes and ionophores place the specificity outside the polymer and already work for ions [102,105]. Synthetic receptors could extend this route to anions, since bis-Schiff base podands have been reported as responsive chemosensors for anions [151]. However, the missing evidence is lifetime data for the membrane rather than for the film. Second, enzymatic and affinity gate functionalisation gives the highest demonstrated specificity and the lowest LODs [103,106]. This performance comes at the cost of the shelf life and temperature range of the biological element. Third, array-plus-model designs move the problem into inference [96,97]. They are the most flexible route and the least auditable. The reason is that a classifier will assign a class to an interferent it has never seen unless it is allowed to abstain [139,141]. Fourth, temperature or bias modulation adds an independent dimension from a single element [98]. It is under-used in conducting-polymer sensing compared with metal oxides, where it is routine. The practical answer is expected to be a combination of three elements. A membrane would exclude the gross interferents, an array would separate what remains, and an abstention rule would mark what neither handles. What would make that combination auditable, and publishable as a standard rather than as a demonstration, is an agreed interference panel measured at an agreed humidity.

Sustainability claims need quantitative support. In a life-cycle assessment of a printed hybrid sensor tag, Zikulnig et al. [152] identified the silicon chip used for data communication as the environmental hotspot. Bio-based polyethylene and copper inks reduced the global warming potential of the tag by up to 39%. Biodegradable conductive-polymer systems and cellulose substrates offer further alternatives [79,153]. Reporting the conductor metal, the substrate, any silicon chip, the service life, and the end-of-life route would let sustainability claims be checked.

This review has four limitations. First, the comparison in Table 9 is assembled from values that studies state explicitly, so it is shaped by reporting practice as much as by physics. Consequently, materials whose community reports LODs routinely will appear stronger than materials whose community does not. Second, this review covers PEDOT:PSS and treats other conducting polymers and composites only as reference points, so conclusions about relative performance are bounded by that scope. Third, the minimum reporting set is argued from the difficulty of comparing the studies cited here. It has not been validated by applying it prospectively to a body of new work. Such a validation is the obvious next step, and one that a single review cannot take. Fourth, several comparisons draw on systems that are not conducting polymers. Examples include the reference rows of Table 7 and Table 8 and the LIG entries of Table 9. The reference rows show the reporting terms and the alternative mechanism, because the quantities concerned were not available within the PEDOT:PSS literature cited here. The LIG entries place the glucose LOD of the OECT route in context. None of these entries is included as a ranking of materials measured under different protocols.

## 11. Conclusions

This review examined PEDOT:PSS as a material for printed flexible sensors and organised the literature around film morphology. Six conclusions follow.

(i) The grain morphology of PEDOT:PSS is the variable to which the largest number of its sensor properties respond. Conductivity, ionic access, mechanics, and water uptake move together, and no treatment changes one of them without the others. However, morphology is not the only variable. Oxidation level, contact resistance, and device architecture act in parallel, and Table 2 marks which links rest on a joint measurement.
(ii) Secondary doping, post-treatment, ionic additives, and crosslinkers offer high conductivity, stretchability, or aqueous stability. However, no single treatment delivers all three.
(iii) The additives that make PEDOT:PSS printable also act as dopants. Print-to-print variation in drying, thickness, and annealing therefore becomes per-unit calibration effort.
(iv) Every transduction mode inherits the sensitivity of PSS to water, which makes humidity the interferent that reaches all of them. It is also the most tractable interferent, because it is reversible and largely common-mode. Slow ageing is largely common-mode as well. The remaining irreversible mechanisms (electrochemical de-doping, cracking, and interfacial debonding) differ by sensor class and do not share a correction. Together with ageing, these mechanisms set the service life.
(v) As a bare chemiresistor, PEDOT:PSS works in the parts-per-million decade, whereas as the channel of an OECT with a functionalised gate, it reaches nanomolar LODs. LOD, linear range, and interference at a stated humidity are rarely reported together.
(vi) Reference channels and drift correction compensate the residual drift that is smooth and common-mode, and only that part. Calibration transfer compensates the unit-to-unit variation that the process leaves, and an abstention rule flags the step changes that no correction removes. In addition, a reporting set that includes humidity and device-wise evaluation makes results comparable.

In summary, the materials toolkit for PEDOT:PSS is mature, and the next advances depend on stability, uniformity, and reporting practice rather than on higher conductivity. 

## Figures and Tables

**Table 4:** Material and device properties measured on the same PEDOT:PSS films.

Change Made to the Film	Material-Level Result	Device-Level Result, Same Films	Stability Measured
Crystallisation with minimised PSS residue [20]	Anisotropic ordering; volumetric capacitance 113 F cm^−3^	OECT transconductance ∼20 mS; $\mu C^{*}\approx$ 490 F cm^−1^ V^−1^ s^−1^	Marginal swelling after >20 days in water; >2000 switching cycles; survives sterilisation
GOPS crosslinking [41]	Conductivity falls but stays high; water uptake and elasticity pass through an optimum	Electrochemical impedance rises	OECTs with 1 wt% GOPS steady over three weeks
Channel thickness at fixed chemistry [42]	Purely volumetric capacitance, 39 F cm^−3^	Transconductance scales with thickness; human brain rhythms recorded	–
Substrate surface treatment and fluorosurfactant [57]	O_2_-plasma skin versus UV/O_3_ plus 1% fluorosurfactant	Resistivity $\times 10^{4}$ at 10% strain versus resistance ×2 at 50%, conductive to 188%	∼2 cracks mm^−1^ at 30% strain; resistivity rises over 1000 stretch cycles
Bilayer passivation over the printed film [61]	Fluorinated and hydrophilic barrier layers added	Temperature sensitivity −0.77% °C^−1^, 25–50 °C	Stable from 30% to 80% RH
Ionic enhancers [38] (counter-example)	>3100 S cm^−1^ at 0% strain; >4100 at 100%	No sensing metric reported	3600 S cm^−1^ after 1000 cycles to 100%; fracture at 800%

**Table 5:** Printing routes for PEDOT:PSS sensors.

Route	Feature Size	Formulation Requirement	Representative Sensing Use
Inkjet	20–100 µm	2–20 mPa s; surfactant for wetting; tuned jetting waveform [64]	Temperature arrays, tattoo electrodes, and gas sensors [48,69,70]
Aerosol jet	10–50 µm	Atomisable dispersion over a wide viscosity range [71]	Planar all-PEDOT:PSS OECT arrays on polyimide [72]
Screen printing	50–100 µm	Thickened paste, 0.5–50 Pa s [62]	Thick electrodes on film and textile
Gravure and flexography	20–100 µm	50–500 mPa s; fast drying [74]	High-volume roll-to-roll layers
Direct ink writing	100–500 µm	Concentrated ink with a yield stress [65]	Three-dimensional electrodes and hydrogels
Related solution-processing route, not printing			
Wet spinning	Fibre diameter	Coagulation bath and post-treatment [53]	Strain-sensing fibres [75]

**Table 6:** Property exploited, interferents, and readout of each PEDOT:PSS transduction mode.

Mode	Property Exploited	Dominant Interferents	Typical Readout
Piezoresistive	Grain contact and film cracking under strain	Humidity, temperature, and viscoelasticity of binder and substrate	Ratiometric bridge with a dummy element
Humidity	Water uptake by the PSS phase	Temperature and slow sorption hysteresis	Resistance or impedance
Temperature	Thermally activated inter-grain transport	Humidity	Resistance behind a humidity barrier
Chemiresistive	Doping change by redox-active gases	Humidity and limited selectivity	Sensor array and classifier
OECT	Volumetric de-doping by cations	Swelling, bias stress, and gate drift	Drain current at fixed bias
Electrophysiological	Volumetric capacitance at the interface	Motion artefact and drying	High-impedance instrumentation amplifier
Thermoelectric	Seebeck coefficient of doped PEDOT	Doping-level drift	Low-noise voltage amplifier

**Table 7:** Mechanical results reported for PEDOT-based films and fibres and for two reference systems.

System	Working Range	Sensitivity or Gauge Factor	Reversibility and Hysteresis	After Cycling
PEDOT:PSS and PEDOT				
Film on O_2_-plasma-treated PDMS [57]	Fails by 10% strain	– (resistivity $\times 10^{4}$ at 10%)	Brittle plasma skin fractures the film	–
Film with 1% fluorosurfactant on UV/O_3_-treated PDMS [57]	Resistance ×2 at 50%; conductive to 188%	–	Buckles after the first stretch above 10% and is then reversible; ∼2 cracks mm^−1^ at 30%, beyond which the film is not reversibly stretchable	Resistivity rises over 1000 stretches
Film with ionic enhancers [38]	To 600%; fracture at 800%	Not reported as a strain sensor	–	3600 S cm^−1^ after 1000 cycles to 100%
Wet-spun polyurethane composite fibre [75]	Elastomeric	– (Young’s modulus rises exponentially with PEDOT:PSS loading)	Reversible and irreversible deformation separated by a filler-network model	–
PEDOT grafted with poly(acrylate-urethane), no PSS [58]	To 500%	–	–	Electrical properties deteriorate with the insulating graft
Topological supramolecular network [40]	High crack-onset strain in a physiological medium	–	–	Stable electromyography (EMG) recordings in vivo
Reference systems, for the reporting terms rather than the material				
Crack-based metallic-nanoparticle film [90]	0.1–7.2%	$2.6\times 10^{8}$ at 7.2% strain	–	–
Screen-printed e-textile conductor [91]	Bending and laundering	–	–	With ironing as a repair step: <20% resistance change after 400,000 bends, <1 Ω after 50 washes

**Table 10:** Failure mechanisms of printed PEDOT:PSS sensors.

Mechanism	Dominant in	Signature in the Signal	Reversible	Common-Mode	Mitigation Reported
Water sorption by the PSS phase	Every mode; the measurand itself for a humidity sensor	Tracks RH within minutes; magnitude set by the PSS fraction	Yes	Yes	Passivation bilayer, stable at 30–80% RH [61]; reference element
Thermal and oxidative ageing	Every dry-film mode	Slow monotonic loss of conductivity over weeks to months	No	Largely	Encapsulation; drift tracked as a time series [36,119]
Over-oxidation and electrochemical de-doping	Transistors and electrodes held under bias	Monotonic loss of transconductance or of injectable charge; depends on bias and duty cycle	No	No	Crystallisation with minimal PSS residue [20]; bias and duty limits
Swelling and delamination in water	Immersed and implanted devices	Step changes and hysteresis on wet–dry cycling	Partly	No	GOPS crosslinking, at the cost of impedance [41]
Cracking within the film	Strain and pressure sensors; any repeatedly bent film	Step increases in resistance; path dependent, not recovered on unloading	No	No	Plasticisers and buckled geometry; working strain kept below crack onset [57]
Debonding at the film–substrate interface	Every device on a flexible substrate	Large step or open circuit, beginning at edges and contacts	No	No	Adhesion layer [81] and neutral-plane placement
Change of contact state at the skin	Electrophysiological electrodes	Low-frequency baseline wander, episodic rather than smooth	Yes, on re-wetting	No	Conformal, self-adhesive designs [110,112]; ionic gel [43]
Laundering and wear	Textile-integrated devices	Resistance change accumulated between uses rather than drift during one; magnitude depends strongly on the protocol [120]	Partly	No	Lamination; domestic ironing as a repair step between cycles [91]

**Table 11:** Minimum reporting set proposed for printed PEDOT:PSS sensors.

Stage	Scope	Report	Otherwise the Reader Cannot
Material	Core	Commercial grade and PEDOT-to-PSS ratio; secondary dopant and concentration; crosslinker; post-treatment solvent, temperature, and time; film thickness and conductivity	reproduce the film, or separate chemistry from processing
Process	Core	Printing route, equipment class, feature size, substrate, and surface treatment; number of devices printed and batch structure	judge whether the result describes a device or a process
Device	Core	Electrode geometry and contacts; encapsulation; excitation (DC or AC, amplitude, frequency)	compare studies, since excitation changes the measured response
Metrology	Core; measured LOD conditional	Full response range; sensitivity with its range of validity; response and recovery times; hysteresis; noise floor; measured LOD	assess whether the figure of merit is usable
Statistics	Core	Number of devices and batches; dispersion of key figures across them	distinguish a material property from a fortunate specimen
Environment	Core	RH and temperature during every measurement; conditioning before testing	separate the measurand from water uptake
Reliability	Conditional on the claimed application	Drift over a stated interval; bending or stretching cycles with radius and strain state; washing protocol	estimate service life
Model	Conditional: only with a trained model	Inputs and model class; device-wise and session-wise data splits; uncertainty or abstention policy	tell generalisation from leakage
Deployment	Conditional: only with a deployment claim	Per-unit calibration procedure, number of paired samples, and recalibration interval; target hardware, latency, and energy	judge the cost and feasibility of deployment
Artefacts	Core	Raw and processed data, analysis code, and any trained models and calibration, with a persistent identifier	reproduce the study

## Data Availability

No new data were created or analysed in this study. Data sharing is not applicable to this article.

## Abbreviations

The following abbreviations are used in this manuscript:

AC	alternating current
ADC	analogue-to-digital converter
BLE	Bluetooth Low Energy
CNT	carbon nanotube
DC	direct current
DMSO	dimethyl sulfoxide
ECG	electrocardiogram
EEG	electroencephalogram
EMG	electromyography
GOPS	3-glycidoxypropyltrimethoxysilane
LIG	laser-induced graphene
LOD	limit of detection
LoRa	long range (low-power wide-area radio)
NFC	near-field communication
OECT	organic electrochemical transistor
OTA	over-the-air
PANI	polyaniline
PDMS	poly(dimethylsiloxane)
PEDOT	poly(3,4-ethylenedioxythiophene)
PEDOT:PSS	poly(3,4-ethylenedioxythiophene):poly(styrene sulfonate)
PSS	poly(styrene sulfonate)
RH	relative humidity
RT	room temperature
UV	ultraviolet
